# Increased fidelity of protein synthesis extends lifespan

**DOI:** 10.1016/j.cmet.2021.08.017

**Published:** 2021-11-02

**Authors:** Victoria Eugenia Martinez-Miguel, Celia Lujan, Tristan Espie--Caullet, Daniel Martinez-Martinez, Saul Moore, Cassandra Backes, Suam Gonzalez, Evgeniy R. Galimov, André E.X. Brown, Mario Halic, Kazunori Tomita, Charalampos Rallis, Tobias von der Haar, Filipe Cabreiro, Ivana Bjedov

**Affiliations:** 1 UCL Cancer Institute, Paul O’Gorman Building, University College London, 72 Huntley Street, London WC1E 6DD, UK; 2 MRC London Institute of Medical Sciences, Du Cane Road, London W12 0NN, UK; 3 Institute of Clinical Sciences, Imperial College London, Hammersmith Hospital Campus, Du Cane Road, London W12 0NN, UK; 4 School of Health, Sport and Bioscience, University of East London, Water Lane, London E15 4LZ, UK; 5 Department of Structural Biology, St. Jude Children’s Research Hospital, 262 Danny Thomas Place, Memphis, TN 38105, USA; 6 Centre for Genome Engineering and Maintenance, College of Health, Medicine and Life Sciences, Brunel University London, London UB8 3PH, UK; 7 Kent Fungal Group, School of Biosciences, Division of Natural Sciences, University of Kent, Canterbury CT2 7NJ, UK; 8 Cologne Excellence Cluster for Cellular Stress Responses in Aging-Associated Diseases (CECAD), University of Cologne, Joseph Stelzmann Strasse 26, 50931 Cologne, Germany; 9 Department of Medical Physics and Biomedical Engineering, University College London, Malet Place Engineering Building, Gower Street, London WC1E 6BT, UK

**Keywords:** ribosome, translation, protein synthesis, aging, mTOR, translation fidelity, translation accuracy, archaea, proteostasis, RPS23

## Abstract

Loss of proteostasis is a fundamental process driving aging. Proteostasis is affected by the accuracy of translation, yet the physiological consequence of having fewer protein synthesis errors during multi-cellular organismal aging is poorly understood. Our phylogenetic analysis of RPS23, a key protein in the ribosomal decoding center, uncovered a lysine residue almost universally conserved across all domains of life, which is replaced by an arginine in a small number of hyperthermophilic archaea. When introduced into eukaryotic RPS23 homologs, this mutation leads to accurate translation, as well as heat shock resistance and longer life, in yeast, worms, and flies. Furthermore, we show that anti-aging drugs such as rapamycin, Torin1, and trametinib reduce translation errors, and that rapamycin extends further organismal longevity in RPS23 hyperaccuracy mutants. This implies a unified mode of action for diverse pharmacological anti-aging therapies. These findings pave the way for identifying novel translation accuracy interventions to improve aging.

## Highlights

• Evolutionarily selected arginine in RPS23 is present only in hyperthermophilic archaea
• RPS23 K60R mutation in flies leads to improved accuracy of protein synthesis with age
• Yeast, worm, and fly RPS23 K60R mutants are longer-lived, healthier, and heat resistant
• Anti-aging drugs, rapamycin, torin1, and trametinib, increase translation accuracy

## Graphical abstract

**Figure:**
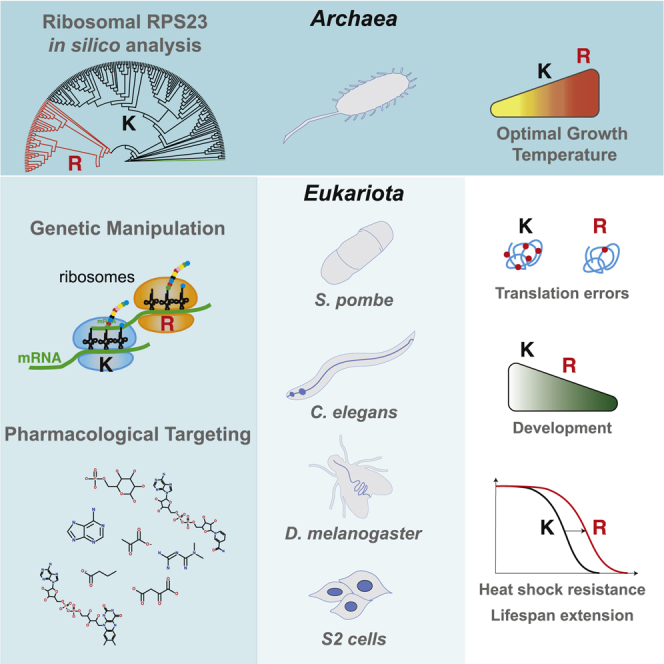

## Teaser

Martinez-Miguel et al. demonstrate that improving translation fidelity by mutating a single amino acid in the decoding center of the ribosome suffices to improve health and longevity in yeast, worms, and flies. This work provides a direct link between fewer errors in translation and longevity.

## Introduction

In stark contrast to the well-established effect of DNA mutations on multi-cellular organismal aging and disease (Garinis et al., 2008), the role of translation errors is far less studied and understood. This is despite mistranslation being the most erroneous step in gene expression. The frequency of protein errors is estimated at 10^−3^ to 10^−6^, depending on the organism and codon (Ke et al., 2017; Kramer et al., 2010; Salas-Marco and Bedwell, 2005; Stansfield et al., 1998). This is several orders of magnitude higher compared to DNA mutations, which are estimated at 1.4 × 10^−8^ per nucleotide site per generation for base substitutions in humans (Lynch et al., 2016). Proteostasis disruption is a critical factor underlying aging and age-related diseases, with translation being one of its key determinants (Hipp et al., 2019; Labbadia and Morimoto, 2015; López-Otín et al., 2013; Steffen and Dillin, 2016). Therefore, an improved understanding of the biological impact of translation errors in the context of organismal aging is very much needed. The role of protein errors in aging was heavily debated in the past (Gallant et al., 1997), mostly due to the lack of causal evidence linking this mechanism to organismal aging. To date, evidence linking translation fidelity and aging is correlative in mammals, and evidence that translation errors are detrimental for aging is exclusively based on single-cell organisms (Anisimova et al., 2018). Recently, the connection between translation fidelity and aging was shown in *Saccharomyces cerevisiae*, where error-prone or ribosomal ambiguity mutants (*ram*) with a point mutation in Rps2 (Rps2 Y143C and L148S) have a shorter chronological lifespan (von der Haar et al., 2017). Similarly, a hypoaccurate mutant in mitochondrial ribosomes of yeast S12 (MRPS12 P50R) has a shorter lifespan, while a hyperaccuracy mutant (MRPS12 K71T) shows extended lifespan and improved cytosolic proteostasis (Suhm et al., 2018). Additionally, slowing down translation elongation by eEF2K-mediated inhibition of eEF2 resulted in improved translation fidelity in mammalian cells *in vitro* (Xie et al., 2019). There is tantalizing evidence from rodent cells, where a correlation exists between translation accuracy and maximum lifespan of different species (Ke et al., 2017). However, translation errors are rarely investigated in the context of multi-cellular organismal physiology, and their effect on aging of metazoan organisms remains unexplored (Rosset and Gorini, 1969). In addition, how to modulate fidelity of protein synthesis to increase lifespan in multi-cellular organisms has not been investigated.

Decoding by the ribosomal accuracy center dictates translation fidelity and is separated into two steps. During the initial tRNA selection, cognate aminoacyl-tRNAs induce domain closure in the small ribosomal subunit, leading to the activation of EF-Tu/EF1A for GTP hydrolysis. In a subsequent proofreading step, the correct aminoacyl-tRNA is inserted into the peptidyl transferase center (Ogle and Ramakrishnan, 2005; Zaher and Green, 2009). Major error contributing factors are misacylation of tRNAs and peptidyl transfer to the mismatched tRNA at the ribosomal A-site (Ogle et al., 2003; Reynolds et al., 2010; Zaher and Green, 2009). We hypothesized that improving fidelity of protein synthesis could be an anti-aging intervention in multi-cellular organisms. Here, we investigated the physiological consequences of directly mutating a single evolutionarily conserved residue in the decoding center of the ribosome and examined for the first time in metazoan species the effect of increased protein synthesis fidelity on aging.

## Results and discussion

### A single substitution in the ribosomal decoding center, RPS23 K60R, reduces stop-codon readthrough translation errors and is evolutionarily conserved in certain archaea

**Figure 1:**
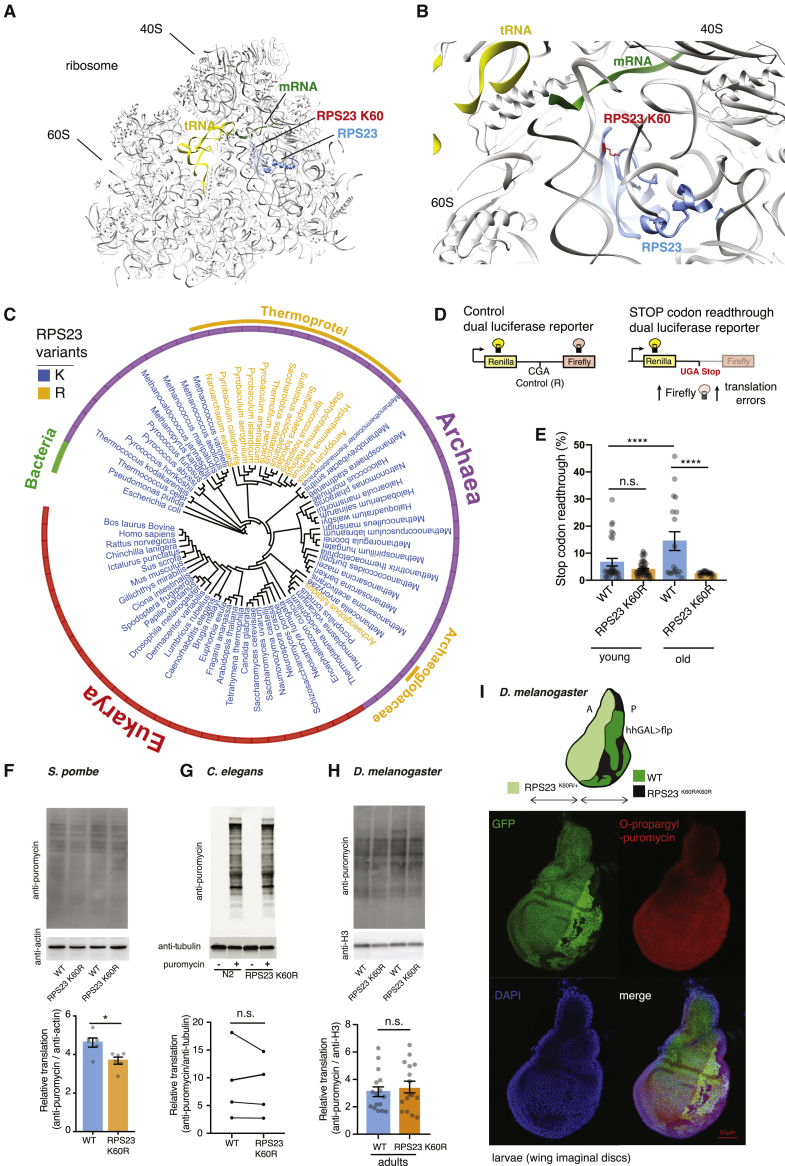
A mutation in the RPS23 (uS12) of the ribosomal decoding center, present in certain thermophilic and hyperthermophilic archaea, improves translation accuracy when introduced to *Drosophila* (RPS23 K60R) (A) Structure of 80S ribosome from rabbit (*Oryctolagus cuniculus*) (Juszkiewicz et al., 2018). (B) A close-up view of the decoding center showing RPS23, lysine residue RPS23 K60, tRNA, and mRNA. (C) Phylogenetic tree of the RPS23 protein sequences from Archaea and Eukarya domains, without branch lengths. *Escherichia coli* is used as outgroup of the tree. The different colors in the outside ring represent the three domains of life, Bacteria, Archaea, and Eukarya. The organism name color denotes amino acid variation of lysine of the conserved KQPNSA region of the RPS23; the organisms in blue have K (lysine) and in orange have R (arginine) residue. The phyla of the organisms with the R variation are represented in the orange outer ring. R variation is the only evolutionarily selected K alternative and is found in some Archaea. (D) Schematic representation of dual luciferase reporters used to assay translation errors in *Drosophila in vivo*. (E) Translation fidelity measurements *in vivo* in young (10-day-old) and old (60-day-old) *Drosophila*. Stop codon readthrough errors in wild-type flies increase with age (p < 0.0001; one-way ANOVA, Tukey’s post hoc test). Fewer STOP codon readthrough errors in old RPS23 K60R mutants versus old wild-type flies (p < 0.0001; one-way ANOVA, Tukey’s post hoc test). No change in stop codon readthrough between young and old RPS23 K60R flies (p = 0.7653; one-way ANOVA, Tukey’s post hoc test). Wild-type young, n = 24; wild-type old, n = 12; RPS23 K60R young, n = 42; RPS23 K60R old, n = 18; from two independent experiments. (F) Translation measurements by *ex vivo* puromycin incorporation and western blotting in *S. pombe* RPS23 K60R mutants and controls during stationary phase (p = 0.011; two-tailed unpaired t tests; n = 6). Quantification of anti-puromycin levels over anti-actin is presented. (G) RPS23 K60R worm mutants do not differ in translation rates compared to N2 controls (p = 0.502; two-tailed paired t test; n = 4). Representative blot of control and puromycin-treated young adult wild-type and RPS23 K60R mutant worms. Quantification of anti-puromycin levels over anti-tubulin. (H) The level of *de novo* protein synthesis was not altered in adult flies with the RPS23 K60R mutation (p = 0.54; two-tailed unpaired t test; n = 16). Quantification of anti-puromycin levels over anti-H3 is presented. (I) Wing imaginal discs in which anterior compartment consists of cells heterozygous for RPS23 K60R mutation, while the posterior compartment consists of wild-type and RPS23 K60R homozygote clones. O-propargyl-puromycin incorporation comparison among genetically different clones shows that RPS23 K60R mutation does not affect translation. hh-GAL4 > UAS-flp stands for hedgehog-GAL4 driven flipase. A, anterior; P, posterior. ^∗^p < 0.05; ^∗∗^p < 0.01; ^∗∗∗^p < 0.001; ^∗∗∗∗^p < 0.0001; n.s., not significant; mean ± SEM.

Structural studies of the ribosomal decoding center in evolutionarily distant organisms point to the importance of the RPS23 protein for translation accuracy due to its role in domain closure and insertion of the aminoacyl-tRNA into the peptidyl transferase center (Figures 1A, 1B, and S1A–S1C) (Loveland et al., 2017; Rodnina et al., 2017; Schmeing and Ramakrishnan, 2009). Indeed, the most well-described hyperaccuracy mutants found in *E. coli* contain mutations in *E. coli*’s RPS23 homolog S12 (Agarwal et al., 2011; Funatsu and Wittmann, 1972; Ogle et al., 2003; Sharma et al., 2007). Therefore, we performed an extensive unbiased phylogenetic analysis of RPS23 in organisms ranging from archaea to eukaryotes, using different databases (see STAR Methods for details), and we have consistently found a lysine residue to be remarkably conserved in the KQPNSA region of ribosomal RPS23, nearly invariant throughout evolution. The only exceptions to this rule are in the thermophilic and hyperthermophilic archea, where the amino acid lysine is replaced by arginine, an event that likely occurred three times independently during evolution (Figures 1C, S1D, and S2A). Analyses of key archaeal characteristics showed that this rare arginine is predominant in archaea that live in extreme conditions such as higher temperatures and acidic environments and that metabolize sulfur. Instead, aerobic and anaerobic metabolism did not discriminate between organisms possessing arginine or lysine in the decoding center (Figures S2B and S2C; Table S1). Moreover, we found that the lysine (K)-to-arginine (R) substitution is an isolated change in RPS23 in this group of organisms, since other regions of the protein are similarly conserved throughout the protein sequence. Therefore, to evaluate the effect of this mutation in higher organisms we focused on this K-R substitution of RPS23 because of its evolutionary presence.

To investigate the link between this single site alteration and translation accuracy, we used CRISPR/Cas9 to introduce a K60R mutation in the KQPNSA region of *Drosophila rps23* (Figures S3A and S3B). To measure translation errors *in vivo*, we created a dual luciferase reporter construct in flies, based on detailed translational studies and accuracy reporters in yeast (Kramer et al., 2010; Salas-Marco and Bedwell, 2005) (Figure 1D). Measurements of stop codon readthrough, which is a common type of translation error (Dunn et al., 2013), showed that in the old RPS23 K60R flies translation accuracy was improved compared to controls (Figure 1E). We also observed that this type of error significantly increased during aging in controls flies, but not in RPS23 K60R mutant (Figure 1E). For the less prevalent misincorporation errors, we did not observe a significant difference between control and RPS23 K60R mutant young flies or old control flies (Figures S3C and S3D), and only a minor significant increase in aged RPS23 K60R flies (Figures S3C and S3D). Thus, unlike stop codon readthrough, misincorporation errors were less frequent and did not increase with age, suggesting that the K60R mutation specifically mitigates age-related translation errors (Figures 1E and S3D).

To investigate the role of the hyperaccuracy mutation in translation rates in evolutionarily distant organisms in addition to *Drosophila*, we introduced the RPS23 K60R mutation in both *Schizosaccharomyces pombe* and *Caenorhabditis elegans* using standard genetic techniques and CRISPR/Cas9, respectively. Next, we measured protein synthesis rates using puromycin, an aminoacyl-tRNA analog that terminates translation and enables detection of nascent polypeptides (Deliu et al., 2017). In yeast, the RPS23 K60R mutation reduced protein translation in a growth phase-dependent manner, with less pronounced effects observed during stationary growth (Figure 1F) compared to exponential growth (Figure S3E). In contrast, puromycin incorporation tests in young adult *C. elegans* showed that the RPS23 K60R mutation did not alter translation (Figure 1G). Similarly, *in vivo* measurements in adult flies showed that global protein synthesis was not affected in RPS23 K60R mutants (Figure 1H). To test if the hyperaccuracy mutation affects translation in rapidly growing and dividing tissues with high protein synthesis demand, we measured translation in the fly larval tissue. To this end, we generated mosaic larval wing imaginal discs. Side-by-side comparison of puromycilated peptides in control and RPS23 K60R heterozygote and homozygote mutant clones in the same tissue clearly showed no alteration in O-propargyl-puromycin incorporation, further suggesting no difference in translation in flies (Figures 1I, S3F, and S3G). Also, the generated mutant clones were of similar size compared to wild-type clones (Figure S3F), showing this ribosomal mutation does not change competitive growth of the cell. These data suggest that the effect of this mutation on decreasing protein synthesis is observable only in single-cell organisms and is not present in multi-cellular metazoans. To exclude non-specific effects on protein translation as a result of the introduction of this genetic modification, we verified that *rps23* gene and protein expression levels remained unaltered in the K60R mutant flies compared to control (Figures S3H and S3I). Finally, we examined additional readouts of altered protein synthesis in flies. We observed no changes between RPS23 K60R mutant and control flies for markers such as phosphorylation of eIF2α (Figure S3J). Similarly, no changes were detected for pS6K or p4E-BP, the downstream effectors of the major regulator of translation mTOR (Figures S3K and S3L).

Overall, we observed a specific reduction of errors in stop codon readthrough in the mutant without an alteration in translation levels between wild-type and RPS23 K60R mutants (Figures 1E and 1H). These findings suggest the translation machinery can accommodate improvements in accuracy without global translation being affected. Given the previously suggested trade-off between translation speed and accuracy (Wohlgemuth et al., 2011), it is interesting that the only hyperaccurate mutation naturally selected by evolution does not impair global translation in metazoans.

### RPS23 K60R mutants in yeast, worms, and flies are heat stress resistant and developmentally delayed

**Figure 2:**
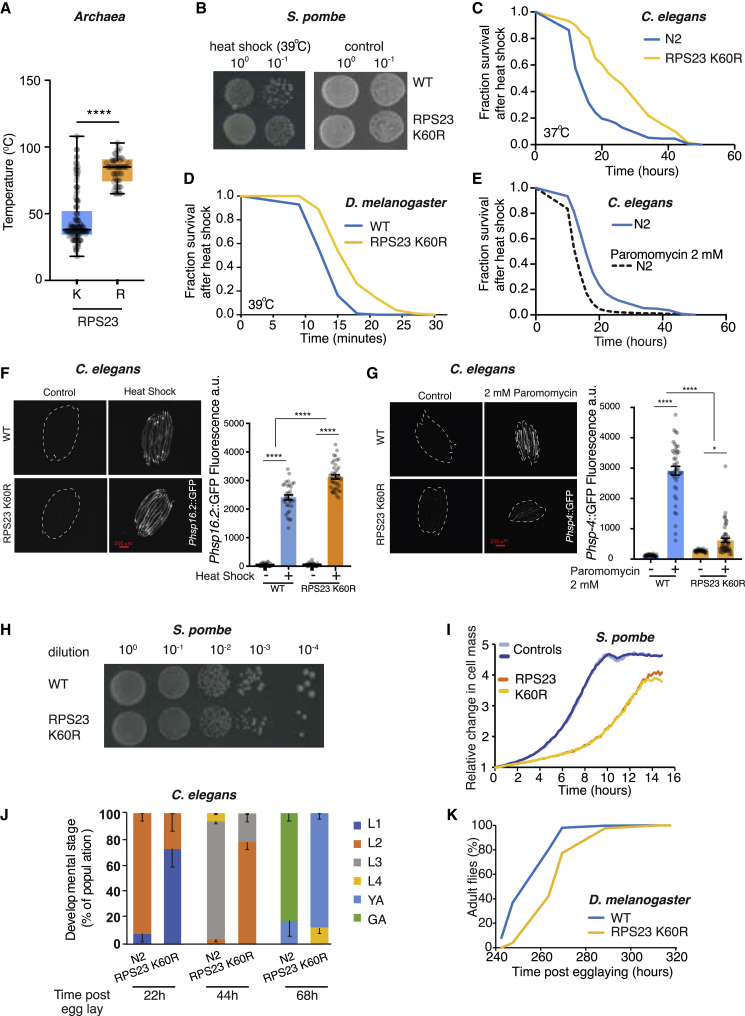
The RPS23 K60R mutants in *S. pombe*, *C. elegans*, and *Drosophila* have enhanced thermotolerance and are developmentally delayed (A) Archaea with arginine (R) instead of lysine (K) in the highly conserved KQPNSA region of RPS23 have higher optimal temperatures (p < 0.0001; two-tailed unpaired t test; K variants, n = 118; R variants, n = 55). Optimal growth temperatures extrapolated from *in vitro* culture measurements of population doubling rates at different temperatures. Data for K and R archaea were obtained from the literature (Table S1). (B) *S. pombe* RPS23 K60R mutant is heat shock resistant. Ten-fold serial dilutions of overnight cultures spotted and heat stressed at 39°C. (C) The RPS23 K60R mutation significantly protects *C. elegans* against the effects of heat shock at 37°C. The survival plot shows the combined survival recovery after heat shock stress of three independent biological replicates (total, n = 153 for wild-type; n = 160 for RPS23 K60R; log-rank test, p < 0.0001). (D) Fly RPS23 K60R mutants are heat shock resistant (39°C; n = 100 for wild-type and RPS23 K60R; log-rank test, p < 0.0001; representative of three independent trials). (E) Paromomycin reduces worm survival upon heat shock stress at 37°C. The survival plot shows the combined survival recovery after stress of three independent biological replicates (n = 247 for wild-type control and n = 244 for wild-type pre-treated with 2 mM paromomycin; log-rank test, p < 0.0001). (F) The RPS23 K60R mutation increases the heat shock response measured by *Phsp-16.2*::GFP upon heat stress. Each image panel on the left shows 10 individual anesthetized worms. Each condition on the right represents 3 independent biological replicates with a total of 33–40 worms. Two-way ANOVA with Tukey’s multiple comparison test, p < 0.0001. (G) An RPS23 K60R mutation significantly protects against the effects of paromomycin on UPR^ER^ activation. Each image panel shows 10 individual anesthetized worms. Each condition on the right represents 3 independent biological replicates with a total of 35–50 worms. Two-way ANOVA with Tukey’s multiple comparison test, p = 0.0227 and p < 0.0001. (H) Decreased growth and smaller colonies of the RPS23 K60R *S. pombe* mutant grown at optimal 32°C. Represented are 10-fold serial dilutions spotted on a YES media plate. (I) Representative growth profiles in microfermentator of RPS23 K60R *S. pombe* mutant compared to control at 32°C. Light and darker colored curves represent two independent biological repeats. (J) Developmental delay of worms with RPS23 K60R mutation. Percentage of animals at defined developmental stages is shown at defined times post parental egg lay. L1–L4 development stages; YA, young adults; GA, gravid adults. Each condition represents 3 independent biological replicates with a total of 50–54 worms. (K) RPS23 K60R mutant flies are developmentally delayed. Wild type, n = 18 vials; RPS23 K60R, n = 15 vials. ^∗^p < 0.05; ^∗∗^p < 0.01; ^∗∗∗^p < 0.001; ^∗∗∗∗^p < 0.0001; n.s., not significant; mean ± SEM.

Next, we sought to investigate the physiological consequences of this mutation. Elevated temperatures and errors in translation are major risk factors for protein misfolding (Balchin et al., 2016; Drummond and Wilke, 2008). Interestingly, propensity for misfolding of erroneous proteins is known to be a major selective pressure driving more accurate protein synthesis (Drummond and Wilke, 2008). Incorporation of erroneous amino acids, particularly in the catalytic site of a protein, could lead to detrimental consequences, and errors in proteins can impose additional energy requirements for folding or protein degradation (Pechmann et al., 2013). Erroneous and misfolded proteins are more prone to damage and aggregation, leading to diminished cellular proteostasis and sensitivity to further insults such as heat stress (Pechmann et al., 2013). This suggests that hyperaccuracy mutants could be more resilient to heat shock. Consistent with this hypothesis, archaea that possess R grow significantly better at higher temperatures than archaea with K (Figure 2A). To probe this hypothesis further, we measured heat stress resistance in all three organisms possessing the RPS23 K60R mutation. Indeed, we observed that the RPS23 K60R mutation resulted in significantly improved survival under heat stress in yeast, worms, and flies, reflecting their improved proteostatic capacity (Figures 2B–2D). Consistent with this interpretation, paromomycin treatment, which increases the error rate in ribosomal translation (Tuite and McLaughlin, 1984), made worms more sensitive to heat shock insult (Figure 2E). To understand the link between translation errors and heat shock response, we used the transcriptional reporters *Phsp-16.2*::GFP and *Phsp-4*::GFP for heat shock (Rea et al., 2005) and endoplasmic reticulum (ER) stress (Ron and Walter, 2007), respectively (Figures 2F, 2G, S4A, and S4B). Induction of *Phsp-16.2*::GFP, which is shown to correlate with longevity (Rea et al., 2005), was more pronounced in RPS23 K60R mutants than in controls upon heat shock, likely contributing to their heat shock resilience (Figure 2C). Further, consistent with the role of paromomycin in specifically producing translation errors, we observed a dose-dependent activation of the ER stress reporter *Phsp-4*::GFP (Figure S4A) to greater levels than induced by heat shock treatment (Figure S4B). Importantly, the K60R mutation significantly protected against ER stress induced by both paromomycin treatment (Figure 2G) and heat shock stress (Figure S4B), suggesting that this ribosomal mutant is protected from insults inducing high levels of proteotoxic stress.

Given these results, we asked why this mutation had not evolved more frequently in nature, given its potential benefit to maintaining a more accurate proteome and making organisms heat stress resilient. A possible explanation could be the existence of negative trade-offs. In agreement with our hypothesis, the RPS23 K60R mutant in *S. pombe* forms smaller colonies (Figure 2H) and shows growth retardation in liquid media (Figure 2I). Similarly, *C. elegans* RPS23 K60R mutants develop slower compared to wild-type controls (Figures 2J and S4C–S4E), have the same size at the last larval L4 stage, are smaller during the reproductive period than day 1 adults (Figures S4E–S4H), and are bigger at the end of the reproductive phase (Figure S4E). In addition, an exhaustive set of measurements of worm behavior, consisting of 2,090 behavioral features, showed that the RPS23 K60R mutation decreases worm size-related features in young day 1 adults, but not other behavioral traits (Figures S4F–S4L; Table S2). Consistent with data from both yeast and worms, *Drosophila* RPS23 K60R mutants were approximately 1 day delayed in eclosing (Figure 2K) and showed delay in pupariation, but the number of flies eclosing was unaffected (Figures S5A and S5B). Additionally, RPS23 K60R flies possess shorter bristles (Figures S5C and S5D) and smaller wings (Figure S5E) and present a very subtle Minute phenotype (Marygold et al., 2007). Overall, these developmental data may explain the presence of the R residue in organisms that live only in extreme conditions for which increased translation fidelity is a strong selective pressure.

### RPS23 K60R is the first metazoan hyperaccuracy mutation that increases lifespan and promotes health

**Figure 3:**
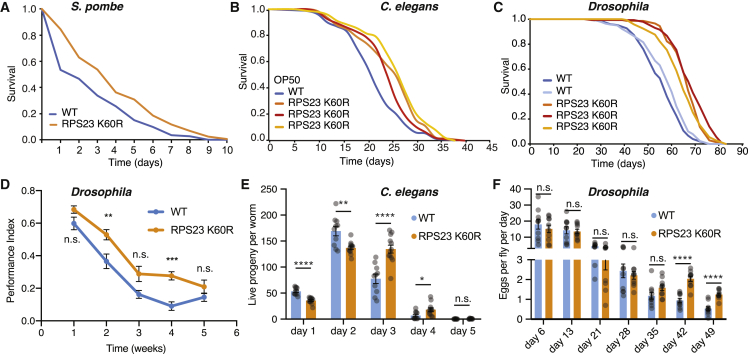
Yeast, worms, and flies with an RPS23 K60R mutation live longer and are healthier (A) *S. pombe* chronological lifespan analysis shows RPS23 K60R mutants live longer compared to controls (p < 0.001; log-rank test). (B) Lifespan analyses showing all three *C. elegans* CRISPR-Cas9 RPS23 K60R lines are longer lived compared to wild-type controls (p < 0.0001; log-rank test; n = 99–144). (C) *Drosophila* lifespan analyses of three independent CRISPR-Cas9 RPS23 K60R lines show they are longer lived compared to both wild-type controls (p < 0.0001; log-rank; n∼150). (D) The RPS23 K60R flies show delayed senescence of negative geotaxis or climbing during aging (two-way ANOVA with Sidak’s multiple comparison test for weeks 1–5; p = 0.3378, p = 0.0051, p = 0.0513, p = 0.001 and p = 0.6409, respectively; n = 10 vials each containing 15 flies). (E) Measurements of the production of live progeny per worm show delayed fertility patterns in RPS23 K60R mutants compared to controls. Multiple unpaired t tests with false discovery rate (FDR) were applied: day 1, q = 0.000014; day 2, q = 0.003167; day 3, q = 0.000237; day 4, q = 0.0017783; day 5, q = 0.049286; RPS23 K60R, n = 12; wild type, n = 10. (F) Fly fecundity measurements for RPS23 K60R mutants and control flies (day 35, q = 0.112219; day 42, q = 0.00002; day 49, q = 0.000049; multiple unpaired t tests with FDR were applied; n = 10 vials of 15 flies). (D and E) Data shown as mean ± SEM.

Collapse of proteostasis is often linked to aging and represents one of its hallmarks (Labbadia and Morimoto, 2015; López-Otín et al., 2013). Therefore, we asked if increased translation fidelity could promote longer life in both single and multi-cellular organisms. Notably, we observed a lifespan extension in RPS23 K60R mutants in yeast, worms, and flies (Figures 3A–3C). The lifespan extension mediated by this single point mutation was 9%–23% in all repeated assays, including *Drosophila* mutants bearing luciferase reporter constructs (Figures 3A–3C and S5F–S5H). In *C. elegans*, the lifespan extension of the RPS23 K60R mutant was equally robust regardless of the bacterial diet. Similar effects on lifespan were observed when worms were grown on standard bacterial food OP50 (Figure 3B) or a relative *E. coli* K-12 BW25113 strain (Figure S5H). Downregulation of translation has a well-established lifespan extension effect (Hansen et al., 2007). Here, by minimally altering the decoding center, we uncoupled increased accuracy from translation downregulation, thereby providing a novel anti-aging intervention.

Long-lived mutant organisms are often healthier with age compared to controls (Kenyon, 2010). To test this premise, we measured fly health during aging using a negative geotaxis or climbing assay. Our data show an overall decline in climbing capacity over time. Importantly, we observed improved climbing of RPS23 K60R flies compared to controls, suggesting healthier aging (Figure 3D). Reduced fecundity is a negative side effect of many long-lived IIS and mTOR mutants (López-Otín et al., 2013), some of which become sterile (Clancy et al., 2001). Also, reproductive senescence is an additional characteristic of aging (Wang et al., 2014). Interestingly, in RPS23 K60R mutant worms we observed fewer progeny being produced at day 1 and 2; however, there was a delayed reproductive decline at day 3 and 4 when worms produced more progeny compared to controls (Figure 3E). Similarly, RPS23 K60R mutant flies also showed slower reproductive decline and produced more eggs at day 42 and day 49, further supporting the idea that adult RPS23 K60R mutants are healthier organisms with age compared to controls (Figure 3F). RPS23 K60R mutation did not affect total number of progeny produced by worms (Figure S5I) or cumulative eggs per fly (Figure S5J). In summary, the ribosomal RPS23 K60R mutation led to improved heat stress resistance and lifespan extension in an evolutionarily diverse range of organisms. This critically highlights the impact of translation fidelity on aging. It reveals for the first time that a direct improvement in translation accuracy by a single amino acid substitution borne out from evolution in the ribosome decoding center extends metazoan lifespan.

### Pharmacological anti-aging interventions, rapamycin, Torin1, and trametinib, reduce translation errors

**Figure 4:**
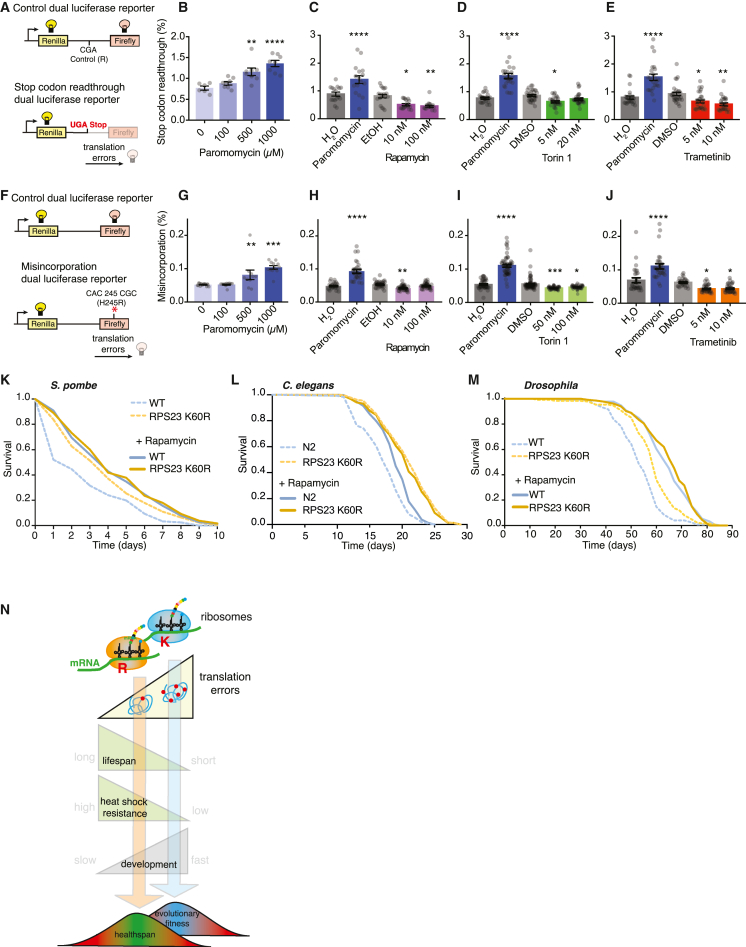
Anti-aging drugs reduce translation errors in *Drosophila* S2R+ cells (A) A scheme of the dual luciferase reporter used to measure stop codon readthrough. (B) The reporter was validated by treating the *Drosophila* S2R+ cells with the error-inducing drug paromomycin (p = 0.0041 and p < 0.0001; control [n = 8] compared to 500 and 1,000 μM paromomycin [n = 6]; one-way ANOVA, Tukey’s post hoc test). (C) Rapamycin-treated S2R+ cells have fewer stop codon readthrough translational errors compared to control cells treated with the respective solvent carrier ethanol (EtOH) (p = 0.0349 and p = 0.0082 for 10 and 100 nM rapamycin versus control; one-way ANOVA, Tukey’s post hoc test; n = 18). (D) 5 nM Torin1 reduces stop codon readthrough translational errors in S2R+ cells (p = 0.0112 for 5 nM Torin1 and p = 0.3184 for 20 nM Torin1 versus DMSO-treated controls; one-way ANOVA, Tukey’s post hoc test; n∼26). (E) Trametinib reduces stop codon readthrough errors (p = 0.0313 and p = 0.0011 for 5 and 10 nM trametinib, respectively, compared to DMSO-treated controls; one-way ANOVA, Tukey’s post hoc test; n∼26). (F) A scheme of the dual luciferase reporter and control reporter used to measure misincorporation translational errors. (G) The misincorporation reporter was validated by treating the S2R+ cells overnight with the error-inducing drug paromomycin (p = 0.0073 and p < 0.0001 for control [n = 17] compared to 500 [n = 10] and 1,000 μM [n = 12] paromomycin; one-way ANOVA, Tukey’s post hoc test). (H) S2R+ cells treated with 10 nM rapamycin have fewer misincorporation translational errors compared to the respective solvent ethanol-treated control cells (p = 0.004 and p = 0.5532 for 10 and 100 nM rapamycin, respectively; one-way ANOVA; n∼35). (I) Torin1 reduces misincorporation translational errors (p = 0.0005 and p = 0.015 for 50 and 100 nM Torin1, respectively, compared to DMSO treatment; n∼56). (J) Trametinib-treated S2R+ cells have fewer misincorporation translational errors (p = 0.044 and p = 0.049 for 5 and 10 nM compared to DMSO; n∼33; one-way ANOVA, Tukey’s post hoc test). (K) Rapamycin extends chronological lifespan in *S. pombe* wild-type (log-rank test, p < 0.001) and RPS23 K60R mutant (log-rank test, p = 0.0267). RPS23 K60R is longer lived than wild-type control in the absence of rapamycin (log-rank test, p < 0.001), but not in presence of 109 nM rapamycin (log-rank test, p = 0.63). (L) Rapamycin treatment at 25 μM extends lifespan of *C. elegans* wild-type worms (p < 0.001; log-rank test; n = 341 and 315 for wild type without and with 25 μM rapamycin). Rapamycin did not extend lifespan of RPS23 K60R mutant (p = 0.4469; log-rank test; n = 283 and 321 for RPS23 K60R without and with 25 μM rapamycin). The survival plot shows the combined survival of 3 independent biological replicates. (M) 100 μM rapamycin treatment extends lifespan of wild-type (p < 0.0001) and RPS23 K60R flies (p < 0.0001). RPS23 K60R is longer lived than wild type (p < 0.0001), albeit not in presence of rapamycin (p = 0.63; log-rank test; n∼150). (N) Schematic representation of the effect of RPS23 K60R hyperaccuracy mutant on lifespan, heat shock stress, and development. Translation accuracy levels that are evolutionarily optimal for fitness are detrimental to organismal longevity.

Great interest in the biology of aging stems from a possibility to improve health in the elderly by mimicking the effect of longevity mutations on organismal physiology through pharmacological approaches (Campisi et al., 2019; Partridge et al., 2018). Interestingly, it was shown that one of the most well-studied anti-aging drugs, the mTOR inhibitor rapamycin (Johnson et al., 2013), reduces errors in translation in mammalian cells *in vitro* (Conn and Qian, 2013; Xie et al., 2019). We explored whether other anti-aging drugs have similar effects on improving translation fidelity. To this end, we adapted our *in vivo* reporters for common translation errors, stop codon readthrough, and amino acid misincorporation for *Drosophila* S2R+ cells (Figures 4A–4J). We validated our reporter systems using the drug paromomycin, which induces translation errors, and observed a dose-dependent increase in errors (Figures 4B and 4G). We showed that, similar to mammalian studies (Conn and Qian, 2013; Xie et al., 2019), rapamycin improved translation fidelity in *Drosophila* S2R+ cells and lowered both stop codon readthrough (Figure 4C) and misincorporation errors (Figure 4H). While the effect of the selective mTORC1 inhibitor rapamycin on aging is extensively studied, effects of dual mTORC1 and mTORC2 catalytic inhibitors are not well explored. We therefore tested the effect of Torin1 on aging. We found that it extends lifespan in *Drosophila* (Figure S6A) (Mason et al., 2018) and, like rapamycin, improves translation fidelity for both types of translation errors (Figures 4D and 4I). Subsequently, we tested trametinib, an MEK/ERK pathway inhibitor, which regulates translation via p90 ribosomal S6 kinase (RSK)-mediated phosphorylation of RPS6 (Roux et al., 2007) and extends lifespan in flies (Slack et al., 2015). Trametinib also improved translation fidelity (Figures 4E and 4J). These findings suggest a novel unifying component in the mechanism underlying anti-aging therapies based on improving translation fidelity. To explore this idea, we tested if the lifespan of RPS23 K60R mutant could be further extended by these pharmacological anti-aging interventions and treated the RPS23 K60R mutant yeast, worms, and flies with rapamycin (Figures 4K–4M). Rapamycin extended lifespan of the wild-type yeast and flies, and to a lesser extent of the long-lived RPS23 K60R mutants, leading to their similar longevity in presence of rapamycin (Figures 4K and 4M). Our results agree with the mechanism of lifespan extension by rapamycin, which is multifactorial and dependent on increased autophagy and lower pS6K (Bjedov et al., 2010) and polIII (Filer et al., 2017). In *C. elegans*, rapamycin extended wild-type lifespan but did not increase the longevity of RPS23 K60R mutant worms further (Figures 4L and S6B). These data suggest a potential higher dependence of worm lifespan on protein fidelity. Overall, the epistasis analysis obtained from the three organisms indicates that when translation accuracy is increased, the capacity of rapamycin to extend lifespan is likely limited to its remaining organism-specific anti-aging components.

### Conclusions

Aging in isolated cultured cells has been studied in the past, but no correlation between errors and aging was found in this context (Anisimova et al., 2018), perhaps owing to insufficient sensitivity of error measurement methods available at the time. In addition, it was shown in yeast that increasing translational errors accelerates loss of viability in yeast (von der Haar et al., 2017). In the context of organismal aging, our data imply that reducing translation errors is an effective strategy for increasing health span. Significantly, these findings add another dimension to our current understanding of the mechanisms of aging, where DNA lesions are often considered a major culprit (Garinis et al., 2008; Tian et al., 2019).

Translation errors, similar to DNA mutations (Mao et al., 1997), may have an adaptive role under stressful conditions (Ribas de Pouplana et al., 2014). Translation accuracy has possibly been selected by evolution to be optimal for adequate cell functioning while enabling rapid, competitive growth and maximal fitness when the environment is favorable (Figure 4N). A strong selection pressure for improved translation accuracy is driven by protein errors that cause protein misfolding (Drummond and Wilke, 2008). Consistent with this view, we show that the naturally occurring RPS23 K60R hyperaccuracy mutation only appears in certain thermophilic and hyperthermophilic archaea, where protein folding needs to occur in physiologically demanding conditions. In contrast, for all other organisms, rapid growth and reproduction are more dominant selective pressures. Therefore, we propose that accurate but developmentally delayed RPS23 K60R mutant organisms would be rapidly outcompeted in the wild.

Historically, there was a general interest in mutations of ribosomal decoding center in single-cell organisms. This particular mutation was previously described in the laboratory settings as hyperaccurate in *E. coli* (Funatsu and Wittmann, 1972) and hypoaccurate in *S. cerevisiae* (Alksne et al., 1993). Here, we show that this single highly conserved amino acid replacement in the decoding center is sufficient to decrease stop codon readthrough errors. Decoding is a complex process, and biochemical and structural studies demonstrate that translation fidelity is affected by interactions and conformational changes of all its interacting partners, the tRNA, rRNA, mRNA, and ribosomes (Zaher and Green, 2009). In an *E. coli* hyperaccuracy mutant rpsL^141^ strain, where an equivalent lysine residue is replaced by asparagine, translation fidelity is mediated through a proofreading step of the tRNA selection process (Zaher and Green, 2010). In our mutant, lysine is replaced with a larger arginine, which provides more stable ionic interactions due to its asymmetrical nitrogen atoms in the guanidium group (Sokalingam et al., 2012). Although this arginine does not directly interact with tRNA or mRNA, rearrangements in the 18s rRNA could propagate to the decoding center. Arginine-induced changes in the rRNA structure could affect the position of mRNA and its interaction with tRNA, leading to increased translation accuracy in the RPS23 K60R mutant. With regard to the mRNA, both its ribose backbone, which is critical for tRNA interactions (Ogle et al., 2001), and phosphodiester bond influencing the mRNA kink structure at the interface of the P and A sites have been shown to affect fidelity of translation (Keedy et al., 2018). Like in bacterial hyperaccuracy mutants, the K60R substitution may disrupt interactions necessary for the closed ribosome conformation (Ogle et al., 2002). Such improvements in translation accuracy lead to advantageous phenotypes including a robust lifespan extension. These effects are observed across taxa, which include multicellular organisms such as worms and flies as well as single-cell organisms such as *S. pombe* fission yeast. Altogether, this suggests the importance of diverse factors such as genetic architecture and environmental conditions in shaping optimal translation accuracy levels. Further exploration of the role played by ribosomal accuracy mutations during healthy aging in diverse biological contexts will be vital to understand its function.

Reduced protein synthesis, either by downregulation of initiation factors or ribosomal proteins (Hansen et al., 2007; Steffen et al., 2008), is a well-established anti-aging intervention. The proposed underlying longevity mechanisms include differential translation (Rogers et al., 2011; Zid et al., 2009), increased Gcn4/Atf4 (Steffen et al., 2008), as well as reduced energy burden to the folding and degradation machinery (Anisimova et al., 2018). Translation can be downregulated and altered during stress in order to allow for production of selected set of proteins (Pizzinga et al., 2020), which could potentially have some shared mechanisms with longevity processes. Despite the reported trade-off between translation efficiency and accuracy (Wohlgemuth et al., 2011), the RPS23 K60R mutation in metazoans improved age-related readthrough accuracy without reducing translation, making our findings distinct from previously reported translation-related longevity mechanisms that are all based on translation downregulation (Anisimova et al., 2018).

A single constitutively expressed misfolding-prone protein is sufficient to compromise the entire cellular proteostasis (Gidalevitz et al., 2006), and reduced translation fidelity through defective editing domain of the tRNA synthetase can cause protein misfolding and neurodegeneration (Lee et al., 2006). Our work demonstrates that increased translation accuracy can be achieved pharmacologically and argues for screening of compounds with the potential to reduce protein errors during aging. Collectively, these findings advocate for the investigation of therapies aiming at increasing translation fidelity in the context of aging and age-related diseases, particularly neurodegenerative diseases that are primarily affected by deterioration of proteostasis (Lee et al., 2006).

### Limitations of study

Our work draws attention to translation accuracy and demonstrates that having fewer protein errors is beneficial for an organism’s resilience to heat stress and longevity. Yet despite careful characterization of hyperaccurate ribosomal mutants in different organisms, it remains to be determined whether these mechanisms are conserved in mammals.

We measured both stop codon and misincorporation errors using the most widely used dual luciferase reporters (Kramer et al., 2010; Salas-Marco and Bedwell, 2005). However, these methods only capture two of the most frequent ribosomal errors. Additional reporters covering a variety of different codons could provide for an in-depth characterization of error prevention conferred by RPS23 K60R ribosomal mutation in diverse physiological contexts. In addition, despite some predictions of the molecular mechanism, based on available ribosomal structures (Keedy et al., 2018; Loveland et al., 2017; Ogle and Ramakrishnan, 2005; Rodnina et al., 2017; Schmeing and Ramakrishnan, 2009; Zaher and Green, 2009), the exact molecular changes in RPS23 K60R mutant ribosomes leading to translation alterations are still elusive and await further investigation.

## STAR★Methods

### Key resources table

**Table:** 

REAGENT or RESOURCE	SOURCE	IDENTIFIER
**Antibodies**		

Anti-puromycin, clone 3RH11	Kerafast	Cat# EQ0001
Anti-puromycin, clone 12D10	Millipore	Cat# MABE343
Anti-tubulin	Sigma-Aldrich	Cat# T6074
Anti-actin	Abcam	Cat# ab8227
Anti-H3	Cell Signaling Technology	Cat# 4499S
Anti-pS6K	Cell Signaling Technology	Cat# 9206S
Anti-total S6K	gift Prof. Linda Partridge laboratory	N/A
Anti- p4EBP	Cell Signaling Technology	Cat# 2855S
Anti-non-phospho-4E-BP	Cell Signaling Technology	Cat# 4923S
Anti- peIF2α	Cell Signaling Technology	Cat# 3398S
Anti- eIF2S1	Abcam	Cat# ab26197
Anti- GAPDH	GeneTex	Cat# GTX100118
Goat anti-mouse IgG	Sigma-Aldrich	Cat# A0168
Anti-rabbit IgG, HRP-linked Antibody	Cell Signaling Technology	Cat# 7074
Anti-mouse IgG, HRP-linked Antibody	Cell Signaling Technology	Cat# 7076

**Bacterial and virus strains**		

*E. coli*: OP50	CGC	RRID: WB-STRAIN:OP50
*E. coli:* BW25113	NBRP	https://shigen.nig.ac.jp/ecoli/strain/resource/keioCollection/list/

**Chemicals, peptides, and recombinant proteins**		

5-Fluoro-2’-deoxyuridine 98+%	Alfa Aesar	Cat# L16497
Agar	Sigma-Aldrich	Cat# A7002
Bacto peptone	BD Biosciences	Cat# 211677
YES Broth	Formedium	Cat# PCM0310
cOmplete EDTA-free protease inhibitor cocktail	Roche	Cat# 11697498001
PhosSTOP EASYpack phosphatase inhibitor cocktail	Sigma-Aldrich	Cat# 4906845001
Dithiothreitol (DTT)	GE Healthcare	Cat# 17-1318-01
LB Broth Miller	Fisher BioReagents	Cat# BP1426
CelLytic Lysis Buffer	Sigma-Aldrich	Cat# C2978
Paromomycin sulfate salt	Sigma-Aldrich	Cat# P5057-1G
Puromycin dihydrochloride	Santa Cruz	Cat# sc-108071B
Puromycin dihydrochloride from *Streptomyces alboniger*	Sigma-Aldrich	Cat# P8833-25G
Laemmli sample buffer 2x	Bio-Rad	Cat# 1610747
Protease inhibitor cOmplete Mini EDTA-free	Roche/Sigma-Aldrich	Cat# 11836170001
Glass beads	Sigma-Aldrich	Cat#G8772
Any kD Criterion TGX Stain-Free Gel	Bio-Rad	Cat#5678123, Cat#5678124
Rapamycin	LC Laboratories	Cat# R-5000
10 mM Tris HCl pH8	Affymetrix	Cat# 22638
1 mM EDTA	Sigma-Aldrich	Cat#EDS-100G
NaCl	Sigma-Aldrich	Cat#S3014
proteinase K	Biotechnology	Cat#E195
brewer’s yeast	MP Biomedical	Cat#903312
nipagin (methyl 4-hydroxybenzoate)	Sigma-Aldrich	Cat#H5501
propionic acid	Sigma-Aldrich	Cat#P1386
Torin1	Tocris	Cat#4247
TRIZOL	ThermoFisher Scientific	Cat#15596026
nuclease-free water	ThermoFisher Scientific	Cat#AM9937
Ambion DNase I kit	ThermoFisher Scientific	Cat#AM2222
ProtoScript II Enzyme mix	New England Biolabs	Cat#E6560S
Power SYBR Green PCR Master Mix	ThermoFisher Scientific	Cat#4367659
Schneider’s *Drosophila* Medium GIBCO	ThermoFisher Scientific	Cat#21720-024
O-Propargyl-puromycin (OPP)	Jena Bioscience	Cat#NU-931-05
Sodium ascorbate	Sigma-Aldrich	Cat#PHR1279-1G
THPTA (tris-hydroxypropyltriazolylmethylamine)	Click Chemistry Tools	Cat#1010
AZDye 568 Picolyl Azide	Click Chemistry Tools	Cat#1292
CuSO_4_	Sigma-Aldrich	Cat#I2852
Vectashield mounting media with DAPI	Vector Laboratories	Cat#H-1200
T4 DNA ligase	NEB	Cat # M0202
Taq DNA Polymerase with Standard Taq Buffer	NEB	Cat # M0273
Q5 High-Fidelity DNA Polymerase	NEB	Cat # M0491
Blasticidin	ThermoFisher Scientific	Cat # A1113903
Penicillin G	ThermoFisher Scientific	Cat # BP2955-5

**Critical commercial assays**		

Clarity Western ECL Substrate	Bio-Rad	Cat# 1705060
Quick Start Bradford Protein Assay Kit	Bio-Rad	Cat# 5000201
Dual Luciferase Assay Reporter Assay System	Promega	Cat# E1910
GenElute Plasmid Miniprep Kit	Sigma-Aldrich	Cat# PLN350
Effectene Transfection Reagent kit	QIAGEN	Cat# 301425

**Experimental models: Cell lines**		

*D. melanogaster*: cell line S2R+	Flybase	FBtc0000150
*D. melanogaster: S2R+* pMT*-* dual luc 868 misincorporation control	This study	N/A
*D. melanogaster: S2R+* pMT*-* dual luc 688 misincorporation H245K	This study	N/A
*D. melanogaster: S2R+* pMT*-* dual luc 690 stop codon control	This study	N/A
*D. melanogaster: S2R+* pMT*-* dual luc 691 stop codon readthrough	This study	N/A

**Experimental models: Organisms/strains**		

*S. pombe h+ ade6-704*	This study	Lab strain KTP126
*S. pombe h+ ade6-704 rps23::TKnatAX*	This study	Lab strain KTP4345
*S. pombe h+ ade6-704 rps23::kanMX6*	This study	Lab strain KTP4359
*S. pombe h+ ade6-704 rps23-K60R::kanMX6*	This study	Lab strain KTP4367
*C. elegans* N2 Bristol	CGC	CGC: N2
*C. elegans* SJ4005 *zcIs4 [hsp-4p::GFP]*	CGC	CGC: SJ4005
*C. elegans* CL2070 *dvIs70 [hsp-16.2p::GFP + rol-6(su1006)]*	CGC	CGC: CL2070
*C. elegans* PHX832 *rps-23(phx832)*	This study	Lab strain PHX832
*C. elegans* PHX833 *rps-23(phx833)*	This study	Lab strain PHX833
*C. elegans* PHX834 *rps-23(phx834)*	This study	Lab strain PHX834
*C. elegans* FGC66 *rps-23(phx833);* 3x backcrossed	This study	Lab strain FGC66
*C. elegans* FGC70 *rps-23(pxh833) dvIs70 [hsp-16.2p::GFP + rol-6(su1006)]*	This study	Lab strain FGC70
*C. elegans* FGC71 *rps-23(phx833) zcIs4 [hsp-4p::GFP]*	This study	Lab strain FGC71
*D. melanogaster y[1]M{w[+mC] = nos-Cas9.P}ZH-2A w[^∗^]*	Bloomington Drosophila Stock Centre	Cat# BDSC 54591
*D. melanogaster* P{ry[+t7.2] = neoFRT}42D	Bloomington Drosophila Stock Centre	Cat# BDSC 1802
*D. melanogaster* w[1118]; P{ry[+t7.2] = neoFRT}42D P{w[+mC] = Ubi-GFP(S65T)nls}2R/CyO	Bloomington Drosophila Stock Centre	Cat# BDSC 5626
*D. melanogaster w[^∗^]; P{w[+mC] = UAS-FLP.D}JD2*	Bloomington Drosophila Stock Centre	Cat# BDSC 4540
*D. melanogaster* hh-GAL4	FlyBase	FBti0017278
*D. melanogaster* hs-FLP	FlyBase	FBtp0000267
*D. melanogaster w*^*Dah*^	gift Prof. Linda Partridge laboratory	N/A
*D. melanogaster w*^*Dah*^*; rps23 K60R*	This study	N/A

**Oligonucleotides**		

Worm *rps-23*_F: GGAAAGCCGAAGGGACTCTGC	N/A	N/A
Worm *rps-23*_R: CTTCTTTCCCTTGAACAGGGCG	N/A	N/A
Yeast TKnatAX_F: agggtttgtgactgttttggacataaag ctaagttcacctaaatccaacacacagttcgccgcaacctc tatactacaaaCGGATCCCCGGGTTAATTAA	This study	N/A
Yeast TKnatAX_R: cggaaaaactacttagactactaaaa ctaatatcattttacgacgcagtaatgagacaaacaactttt tattaagttcgtGAATTCGAGCTCGTTTAAAC	This study	N/A
Yeast rps23-AatII_F: aatgcaaga cgtcTCTTCGGCAGAACTTTCGTC	This study	N/A
Yeast rps23-AscI_R: aatgcaaggcgc gccAGCAAAGAGTCTGACACAGG	This study	N/A
Yeast rps23 verification_F: TCTTCGACTGCTTCCTCTTC	This study	N/A
Yeast rps23 verification_R: TAAGAAGGGTAGGGTTTGTGAC	This study	N/A
Yeast rps23 verification2_F: aatgcaa gctagcAGCTCTAGGCTTTTCCTTCTT	This study	N/A
Yeast rps23 verification2_R: ACCCTCTTTCACTTCTCCAG	This study	N/A
Fly rps23 gDNA_F: GTCGCT ACCGTCACGGGGCACGA	This study	N/A
Fly rps23 gDNA_R: AAACTCG TGCCCCGTGACGGTAG	This study	N/A
Fly pCDF3U6-rps23-gRNA_F: GTCGCT ACCGTCACGGGGCACGA	This study	N/A
Fly pCDF3U6-rps23-gRNA_F: GTTCG CTTAATGCGTATGCA	This study	N/A
Fly ssODN rps23-K60R: ATATGATATCAATTATA TTAATCTCTTAGTGGTATATCAAAACTAATCGG TTTCCTCTACTCCACAGCGGCGTCGAGGCCC GCCAGCCCAACTCAGCCATCCGCAAGTGCGT GAGGGTGCAGCTGATCAAGAACGGCAAGAAG ATCACCGCCTTCGTGCCCCGTGACGGTAGCT TGAACTACAT	This study	N/A
Fly rps23-K60R PvuII verification F: CGACAAGGACTACAAGAAGG	This study	N/A
Fly rps23-K60R PvuII verification R: TGCTTGTCTGGAAAAAGATT	This study	N/A
Fly rps23-K60R mutant only amplification F: GTCCGAAAATCGCACAAAATCCAG	This study	N/A
Fly rps23-K60R mutant only amplification R: GGCTGAGTTGGGCTGGCG	This study	N/A
Fly RT-qPCR actin5C F: GAGC GCGGTTACTCTTTCAC	This study	N/A
Fly RT-qPCR actin5C R: GCCATC TCCTGCTCAAAGTC	This study	N/A
Fly RT-qPCR rps23 F: CGCTTC AAGGTTGTCAAGGT	This study	N/A
Fly RT-qPCR rps23 R: AGATCTT GGGCGTTCCTTCT	This study	N/A

**Recombinant DNA**		

pFA6a-kanMX6	Amelina et al., 2016	N/A
pCFD3-dU6:3g	Addgene	Cat#49410
pDB868 (misincorporation control)	(Salas-Marco and Bedwell, 2005)	N/A
pDB688 (misincorporation H245K)	(Salas-Marco and Bedwell, 2005)	N/A
pDB690 (stop codon control)	(Salas-Marco and Bedwell, 2005)	N/A
pDB691 (stop codon readthrough UGA*C*)	(Salas-Marco and Bedwell, 2005)	N/A
pUAST-attB-Ub-dual luc 868	This study	N/A
pUAST-attB-Ub-dual luc 688	This study	N/A
pUAST-attB-Ub-dual luc 690	This study	N/A
pUAST-attB-Ub-dual luc 691	This study	N/A
pENTR3C	Thermo Fisher Scientific	Cat# A10465
pMT	Addgene	Cat# 17923
pMT- dual luc 868	This study	N/A
pMT- dual luc 688	This study	N/A
pMT- dual luc 690	This study	N/A
pMT- dual luc 691	This study	N/A

**Software and algorithms**		

R (v3.5.0)	R Core Team	https://www.r-project.org
Python (v3.6.10)	Python Core Team	https://www.python.org
GraphPad Prism 8	GraphPad Software	https://www.graphpad.com/scientific-software/prism/
JMP 14	SAS Institute	http://www.jmp.com/en_be/software/data-analysis-software.html
FIJI (v1.53c)	FIJI- ImageJ	https://imagej.net/software/fiji/
Zen 2 (Blue edition)	Zeiss	https://www.zeiss.com/microscopy/int/products/microscope-software/zen-core.html
Tierpsy Tracker software (version 1.5.2)	Andre E. X. Brown (Javer et al., 2018)	https://github.com/ver228/tierpsy-tracker/releases
mafft (v7.460)	(Nakamura et al., 2018)	https://mafft.cbrc.jp/alignment/software/
IQ-TREE (v1.6.9)	(Minh et al., 2020)	http://www.iqtree.org/
ggtree (v2.1.1)	(Yu et al., 2017)	https://bioconductor.org/packages/release/bioc/html/ggtree.html
PhyloT and iTOL	Letunic and Bork, 2019	https://phylot.biobyte.de/

**Other**		

Drosoflipper device for fly transfer; http://www.drosoflipper.com/	Scientific Laboratory Supplies	FLY1386
Flybase	N/A	http://flybase.org
NCBI RefSeq protein database	N/A	https://www.ncbi.nlm.nih.gov/refseq/

### Resource availability

#### Lead contact

Further information and requests for resources and reagents should be directed to and will be fulfilled by the Lead Contact, Ivana Bjedov (i.bjedov@ucl.ac.uk).

#### Materials availability

Plasmids and strains generated in this study will be made available upon reasonable request to the Lead Contact Ivana Bjedov (i.bjedov@ucl.ac.uk).

### Experimental model and subject details

#### Yeast strains

Yeast strain *S. pombe h+ ade6-704* is a stock from Kazunori Tomita laboratory (strain KTP126). Other strains, KTP4345 *h+ ade6-704 rps23::TKnatAX,* KTP4359 *h+ ade6-704 rps23::kanMX6, and* KTP4367 *h+ ade6-704 rps23-K60R::kanMX6* are from this study. Strains were generated using standard genetics and cloning techniques as described in detail below.

#### Worm strains

*C. elegans* N2 Bristol (wild-type) strain was obtained from the *Caenorhabditis* Genetics *Center* (CGC). *C. elegans* SJ4005 *zcIs4 [hsp-4p::GFP]* and CL2070 *dvIs70 [hsp-16.2p::GFP + rol-6(su1006)]* are also from the CGC. Using the CRISPR/Cas9 system at SunyBiotech this study generated *C. elegans* strains: PHX832 *rps-23(phx832),* PHX833 *rps-23(phx833)* and PHX834 *rps-23(phx834),* details of strain construction are described below. This study also generated following strains: FGC66 *rps-23(phx833)(*3x backcrossed), FGC70 *rps-23(pxh833) dvIs70 [hsp-16.2p::GFP + rol-6(su1006)],* and FGC71 *rps-23(phx833) zcIs4 [hsp-4p::GFP]*.

#### Fly strains

The *Drosophila melanogaster white Dahomey* (*wDah*) wild-type strain used in this study was collected in 1970 in Dahomey (now Benin) and has since been maintained in large population cages with overlapping generations. From Bloomington *Drosophila* Stock Centre, we acquired BDSC 54591 *y[1]M{w[+mC] = nos-Cas9.P}ZH-2A w[^∗^],* BDSC 1802 P{ry[+t7.2] = neoFRT}42D, BDSC 5626 w[1118]; P{ry[+t7.2] = neoFRT}42D P{w[+mC] = Ubi-GFP(S65T)nls}2R/CyO, BDSC 4540 *w[^∗^]; P{w[+mC] = UAS-FLP.D}JD2.* Stocks described in Flybase were FBti0017278 hh-GAL4 and FBtp0000267 h-FLP. In this study we generated *w*^*Dah*^*; rps23 K60R* using CRISPR/Cas9, and details of strain construction are described below.

#### Yeast growth condition

All media and supplements were purchased from FORMEDIUM. Fission yeast were maintained by growing at 32°C with constant shaking.

#### Worm growth condition

Worms were maintained at 20°C, unless otherwise stated, on nematode growth medium (NGM) seeded with *E. coli* strain OP50 except in Figure S5H where strain BW25113 was used. For rapamycin treatments, rapamycin was dissolved in (90:10) ethanol-tween 20 at 50 mg/mL by vigorous vortexing. The rapamycin stock was diluted in molten NGM to obtain the desired concentration. Control plates were obtained by dissolving an equal volume of (90:10) ethanol-tween 20. Plates were dried and seeded with *E. coli* OP50 and kept for 24 h at 20°C before adding L1 worms.

#### Fly growth condition

All experiments were conducted using *white Dahomey* (*w*^*Dah*^) wild-type flies that were maintained at 25°C. Flies were kept on a 12 h light:12 h dark cycle at constant humidity using standard sugar/yeast/agar or SYA media (Bass et al., 2007) (sugar 50 g/l; yeast 100 g/l (MP Biomedical; 903312); agar 15 g/l (Sigma; A7002); supplemented with nipagin 30 mL/L (Sigma H5501) of 10% w/v in 95% EtOH and propionic acid 3 mL/l (Sigma P1386) as antifungal agents). For Torin 1 longevity analysis, we used holidic media recipe food to increase drug bioavailability (Piper et al., 2014). Torin 1 concentration in holidic media for fly lifespan experiments was 10 μM (Tocris, 10 mM stock dissolved in DMSO). Rapamycin concentration in SYA fly food for longevity analysis was 100 μM (LC Laboratories, 50 mM stock dissolved in ethanol).

#### S2R+ cells growth condition

*Drosophila* S2R+ cells were cultured in Schneider’s *Drosophila* medium (ThermoFisher, GIBCO 21720-024) supplemented with 10% heat inactivated fetal bovine serum (FBS) at 25 C.

### Method details

#### Structural modeling

For structural modeling we used the published Protein Data Bank (PDB) structure of the 80S ribosome stalled on globin mRNA at the stop codon obtained from the rabbit *Oryctolagus cuniculus*; https://doi.org/10.2210/pdb6HCF/pdb EMDataResource: EMD-0192 (Juszkiewicz et al., 2018). For supplementary structural modeling we used ribosome structures form archaea *Pyrococcus abyssi;* https://doi.org/10.2210/pdb6SWC/pdb EMDataResource: EMD-10322 (Coureux et al., 2020), yeast *Saccharomyces cerevisiae* ribosome structure; https://doi.org/10.2210/pdb5M1J/pdb EMDataResource: EMD-4140 (Hilal et al., 2016), and human *Homo sapiens*; https://doi.org/10.2210/pdb6HCF/pdb EMDataResource: EMD-0192 (Juszkiewicz et al., 2018).

#### Phylogenetic analysis

All RPS23 sequences from Archaea and Eukarya displayed in Figure S1 were downloaded from Interpro. Sequences larger than 200 amino acids were removed from the analysis. Sequences in Figure 1C used only curated entries obtained from Swiss-prot. For multiple sequence alignment we used MAFFT software with auto mode. Trees were generated based on multiple alignment using IQ-TREE, a phylogenomic inference software. ModelFinder was used to determine the best protein model for the trees (LG+G4 for the tree in Figure 1C and LG+R8 for the tree in Figure S1). Two bacterial species, *Escherichia coli* and *Pseudomonas putida*, were used as outgroup. Tree representation and annotation was done with ggtree, treeio, and tidytree R Libraries.

Sequences used in Figure S2 were obtained from the NCBI RefSeq protein database: research query: 30S ribosomal protein S12 (option: archeae only). Sequences removed from the dataset include all non-30S ribosomal protein S12, MULTISPECIES 30S ribosomal protein S12, and duplicated sequences for the same organism when sequences have the same mutation of interest (i.e., K or R). Alignment was performed using Geneious Prime with MAFFT alignment auto: FFT-NS-I method. Tree generation was based on multiple alignment: IQ-TREE parameters: The best-fit model chosen to create the tree was LG + R6 according to the Bayesian Information Criterion. Two bacteria were used as outgroup, *Escherichia coli* and *Pseudomonas putida*.

Tree generation was based on NCBI taxonomy PhyloT online tool tree annotation: iTOL (interactive Tree Of Life) (Letunic and Bork, 2019) (available at https://phylot.biobyte.de/). For multiple correspondence analysis and hierarchical clustering we used the following software R studio packages: FactoMineR, factoextra, FactoInvestigate and explor. Clustering was achieved using the MCA and HCPC (nclust = 2) methods. For more information please see Kalyaanamoorthy et al. (2017) and Katoh et al. (2019).

#### Generation of RPS23 K60R mutant in *S. pombe*

The TKnatAX cassette (Amelina et al., 2016) was amplified using primers with the RPS23 overhangs: (agggtttgtgactgttttggacataaagctaagttcacctaaatccaacacacagttcgccgcaacctctatactacaaaCGGATCCCCGGGTTAATTAA and cggaaaaactacttagactactaaaactaatatcattttacgacgcagtaatgagacaaacaactttttattaagttcgtGAATTCGAGCTCGTTTAAAC). The respective PCR product was used to transform *S. pombe* and the positive candidates, which had the RPS23 gene replaced by the TKnatAX cassette, were detected using PCR. The Rps23 gene was cloned into the pFA6a-kanMX6 cassette using AatII and AscI restriction sites (oligos for amplification of rps23 were aatgcaagacgtcTCTTCGGCAGAACTTTCGTC and aatgcaaggcgcgccAGCAAAGAGTCTGACACAGG). The K60R mutation was introduced using site directed mutagenesis. The mutated RPS23 K60R was cut out from the pFA6a-kanMX6 plasmid using SalI and SacI enzymes, gel purified, and used to transform *rps23*Δ (*rps23*::TKnatAX). Homologous recombination allowed replacement of TKnatAX cassette with the rps23 mutant allele (along with the kanMX6 cassette). Positive candidates containing the RPS23 K60R mutation were selected on FdU plates for further experiments. The PCR verification primers used were TCTTCGACTGCTTCCTCTTC and TAAGAAGGGTAGGGTTTGTGAC or aatgcaagctagcAGCTCTAGGCTTTTCCTTCTT and ACCCTCTTTCACTTCTCCAG.

#### *S. pombe* growth assay

Fission yeast were maintained by growing at 32°C. Growth curves were automatically determined using the BioLector microfermentation system (m2p-biolabs), using 48-well flowerplates with 1.5 mL of media, as previously described (Rallis et al., 2013). Rapidly growing cultures at OD_600_ = 0.5 in Yeast Extract with Supplements (YES) were diluted to OD_600_ = 0.15, plated in flowerplate wells and incubated at 32°C with constant shaking at 1000 rpm. Cell growth was monitored by recording light scattering every ten minutes for 15 h. Relative mass increase was calculated by normalizing to starting (time zero) values.

#### *S. pombe* chronological lifespan assay

Cells were grown in YES as previously described (Rallis et al., 2013). When cultures reached a stable maximal density, cells were harvested, serially diluted, and plated on YES plates. The measurement of colony-forming units (CFUs) was taken as time point 0 at the beginning of the CLS curve (i.e., 100% cell survival). Measurements of CFUs were conducted on the following days until cultures have diminished cell survival. Error bars represent standard deviation calculated from three independent cultures, with each culture measured three times at each time point. Survival curves were statistically analyzed by comparing AUCs measured by FIJI (Schindelin et al., 2012) coupled with t tests.

Chronological lifespans in the presence of rapamycin were performed as previously described in Rallis et al. (2013). In summary, fast growing wt and RPS23 K60R mutant cells were treated with 100 ng/mL rapamycin at OD_600_ = 0.2. As in the case of the cultures without rapamycin colony forming units (CFU) were determined for each day following entrance to stationary phase.

#### *S. pombe* heat shock assay

Saturated overnight cultures were serially diluted and 10 μL of each dilution was plated on YES media plate. After spots were absorbed, each plate was placed at 39°C for 3 days and then at 32°C for one day recovery before counting colony forming units and imaging.

#### Translation measurement using puromycin incorporation assay and western blot in *S. pombe*

*S. pombe* were grown at 32°C with shaking to exponential (OD_600_ = 0.45) or stationary phase (OD_600_ = 6.5) before a 30 min treatment with 10 μM puromycin (Sigma; P8833). Samples were then centrifuged and the pellet was frozen. For western blot sample preparations, the pellet was diluted in Laemmeli sample buffer (Bio-Rad; 1610747) containing 50 mM DTT and protease inhibitor (cOmplete Mini EDTA-free; Roche) cocktail. Glass beads (Sigma, G8772) were added and cells were lysed in a Fastprep-24 machine (MP Biomedicals). Approximately 20 μg of protein extract was loaded on a precasted Any kD TGX stain-free gels (Bio-Rad; 567-8123 or 567-8124). Proteins were separated and transferred to a nitrocellulose membrane using wet transfer. Blots were developed using the ECL detection system (Clarity Western ECL Substrate; Bio-Rad; 1705060), imaged using CCD camera system of ImageQuant LAS 4000 (GE Healthcare), and analyzed using FIJI software (US National Institutes of Health). Antibodies used were anti-beta actin (Abcam; ab8227; 1:2000) and anti-puromycin [3RH11] (Kerafast; Equation 0001; 1:2000).

#### Worm RPS23 K60R mutant strain generation

3 independent *rps-23* (F28D1.7) mutant lines (PHX832, PHX833 and PHX834) were generated at SunyBiotech using the CRISPR/Cas9 system by mutating aag to cga in the KQPNSA region. The presence of the (K60R) mutation in each strain was confirmed by performing single worm PCR using the primer pair *rps-23*_F: GGAAAGCCGAAGGGACTCTGC and *rps-23*_R: CTTCTTTCCCTTGAACAGGGCG in both genomic DNA from wild-type and *rps-23* mutants to generate a 685bp fragment. The PCR product was treated with Blp I enzyme for 1 h at 37C. Electrophoretic separation of the digested PCR products obtained from amplication of the genomic material from *rps-23* produces one band and two bands from the mutant and wild-type, respectively. The *rps-23* mutant strain FGC66 used throughout this study, was obtained by backcrossing PHX833 three times to our laboratory N2 wild-type CGCH strain, formerly obtained from the Caenorhabditis Genetics Center. The FGC66 strain was crossed with strain CL2070 to generate FGC70 *rps-23(K60R) dvIs70 [hsp-16.2p::GFP + rol-6(su1006)]* and with SJ4005 to generate FGC71 *rps-23(K60R) zcIs4 [hsp-4p::GFP]*.

#### Worm development assays

30-40 N2 and RPS23 K60R mutant day 1 adults grown on OP50 bacteria were transferred to NGM plates seeded with OP50 to lay eggs for 3 h. Parents were removed and the progeny was allowed to develop for 51 h at 20°C. The progeny was washed from the plates and the length and extinction of each worm was measured using COPAS Biosorter equipped with LP Sampler (Union Biometrica; Holliston, MA). The gate for the L4 developmental stage was set by measuring hand-picked L4 worms using the COPAS Biosorter. The percentage of worms inside and outside the gate were determined per genotype/condition over the total number of worms measured. Each data point for each condition represents over 100 individual worms from an independent biological replicate. 3 independent experimental trials were performed per genotype.

For size assays performed by microscopy, 50 N2 and RPS23 K60R mutant day 1 adults grown on OP50 bacteria were transferred to NGM plates seeded with OP50 to lay eggs for 2 h and killed after. Progeny were randomly taken from NGM plates at 22 h, 44 h and 68 h. For staged L4, day 1 and day 4 adult size assays, L4-staged worms for each genotype were handpicked to freshly seeded NGM plate and kept for 4 days with regular transfer every day to freshly seeded plates. Worms for each genotype were selected for imaging at the L4 stage, and as day 1 and day 4 adults. Imaging was performed on anaesthetized worms with 2% levamisole hydrochloride under a 63x objective using a Zeiss Axio Zoom V16 microscope system equipped with an AxioCam MRm camera operated by Zen 2 software (Zeiss). All images were exported in TIFF or CZI format and sizes were quantified using FIJI on a Surface tablet (Microsoft). 3 independent experimental trials were performed with each one containing at least 10 worms per genotype per time point.

#### Worm transgenic reporter assays

The following strains were used for these assays: CL2070 *dvIs70 [hsp-16.2p::GFP + rol-6(su1006)]*, FGC70 *rps-23(K60R) dvIs70 [hsp-16.2p::GFP + rol-6(su1006)]*, SJ4005 *zcIs4 [hsp-4p::GFP]* and FGC71 *rps-23(K60R) zcIs4 [hsp-4p::GFP]*. For paramomycin assays, paramomycin was added directly to molten NGM agar to obtain a final concentration of 0.5, 1 and 2 mM. Plates were kept at 4°C until needed. Plates were dried and seeded with UV-irradiated *E. coli* OP50. L4-staged worms from each genotype were placed in no drug or paramomycin plates for 48 h at 20°C before imaging. For heat shock measurements of strains CL2070 and FGC70, day 2 adult worms were heat shocked for 6 h at 30°C before imaging. For heat shock measurements of strains SJ4005 and FGC71, day 2 adult worms were heat shocked for 4 h at 37°C before taking images. Imaging was performed on anesthetized worms with 2% levamisole hydrochloride under a 63x objective using a Zeiss Axio Zoom V16 microscope system equipped with an AxioCam MRm camera operated by Zen 2 software (Zeiss). The GFP filterset (excitation: 450-490 nm; emission: 500-550 nm) was used. All images were exported in TIFF or CZI format and fluorescence levels were quantified using FUJI run on a Surface tablet (Microsoft). The fluorescence intensity of individual worms was calculated as the pixel density of the entire cross-sectional area of the worm from which the pixel density of the background had been subtracted. 3 independent experimental trials were performed with each one containing at least 10 worms per genotype per time point.

#### Worm reproductive assays

Wild-type N2 and RPS23 K60R mutants were grown on OP50 until the L4 stage. Individual L4 worms were placed in 1 day-old seeded OP50 plates and transferred every day for 6 days to freshly seeded plates. Progeny per worm per day per genotype were counted after 24 h of the transfer of the parent worm. 3-4 worms were measured per genotype and 3 independent experimental trials were performed per genotype.

#### Worm heat shock survival assays

Wild-type N2 and RPS23 K60R mutant adults were grown and aged to day 4 by transferring every day to fresh NGM plates seeded with *E. coli* OP50. On day 4 of adulthood plates were wrapped with parafilm and submerged in a water bath at 37°C for 3 h. For the paramomycin heat shock experiment, worms were aged to day 2 before transferring to NGM plates containing 2 mM paramomycin and seeded with UV-irradiated bacteria for 24 h. On day 3 of adulthood, plates were wrapped with parafilm and placed in a water bath at 37°C for 4 h. After heat shock, plates were transferred to an incubator at 20°C and scored throughout their entire lifespan at the indicated time points. Animals were scored dead if they didn’t respond to touch with a pick. 3-5 independent experimental trials were performed per genotype with at least 50 animals per trial.

#### Worm behavioral assays

Wild-type N2 and RPS23 K60R mutant worms were grown on NGM *E. coli* OP50 plates till reaching the first day of adulthood. One worm per well was handpicked to a 96-square well plate containing NGM and freshly seeded with OP50. 1 h prior to imaging, 96-well plates were placed in the imaging cave to acclimate at 20°C. The plates were recorded under the imaging rig for 15 min at four time points: 2, 4, 6 and 24 h after worms were picked onto them. Each 15-min recording was composed of three consecutive 5-min videos, termed pre-stimulus, blue-light, and post-stimulus. Each 5-min stimulus video was analyzed independently to investigate worm behavior before, during and after delivering a blue-light stimulus to the worms, respectively. Worms were exposed to blue-light emitting diodes to expand the range of behaviors observed in the assay, as they produce a sufficiently bright light to induce an escape response in the worms, thus expanding the phenotypic space for observed behavioral differences.

Videos were analyzed using Tierpsy Tracker software (version 1.5.2), which segments, tracks and skeletonizes the worms in the videos, and extracts a quantitative set of features that capture behavioral differences and can be used to discriminate between RPS23 K60R mutant and wild-type *C. elegans* (Yemini et al., 2013). Summary statistics for a total of 3016 features were computed for each well, as an average of the worm present in the well over the 5-min period (Javer et al., 2018). Considering behavior separately before, during and after exposure to blue-light, this yielded a total of 9048 features for the analysis. Pre-stimulus videos were manually inspected using Tierpsy’s Well Annotator GUI to identify wells with poor quality agar, contamination, or no worms in them, and exclude their feature summary results from the analysis. 91 wells in total were omitted. To eliminate noise in the data from erroneously tracked objects of similar size and shape to worms, for example due to contaminants or reflections on the agar, a background subtraction step was first performed to segment objects that move between frames in the video. A trained CNN classifier was then used to identify worm-like objects, and a threshold filter was applied to calculate feature summaries for tracked objects with a recorded length between 200 and 2000 μm, and midbody width between 20 and 200 μm (resolution: 12.4 μm/pixel). These steps ensured that no non-worm object was analyzed. Feature summary results were then further cleaned to remove features that were recorded to have zero standard deviation (4 features), or more than 20% missing values (335 features, 3.7%). Remaining missing values were imputed using the global mean value for each feature (9.4% of data). An additional 480 features related to path curvature were also omitted due to noise in their computed summary statistics. Finally, summary statistics for the middle 50^th^ percentile of the distribution for each feature were chosen, yielding a reduced set of 2090 features that were used for the analysis. 93% of features were found to be normally distributed (Shapiro-Wilks normality test, p > 0.05).

The experiment was repeated on three separate dates with 48 animals per genotype, and a linear mixed model was performed to test for significant features between K60R mutant and N2 control, after accounting for day-to-day variation as a random effect and controlling the false discovery rate at 5% with the Benjamini-Yekutieli correction for multiple comparisons. Significant differences were observed at all imaging time points in at least 3% of features (LMM, p < 0.05). 6 h was selected as the optimal time point for observing differences between RPS23 K60R and N2, as by this time worms have had ample time to settle on the assay plates and assume normal feeding behavior. After 6 h, a total of 72 (3.4%) features for RPS23 K60R were found to be significantly different (LMM, p < 0.05). All presented data are reported for the 6 h time point.

All statistical analyses and visualization were performed in Python v3.6.10 using the following notable packages (numpy v1.18.4, pandas v0.25.3, scipy v1.4.1, statsmodels v0.11.1, scikit-learn v0.23.0, pytorch v1.4.0, matplotlib v3.2.1, and seaborn v0.11.0). GitHub: https://github.com/saulmoore1/PhD_Project.git. Details of behavioral analysis are provided in Table S2.

#### Worm lifespan assays

Lifespan measurements were performed as follows. Axenic worm eggs for wild-type or *rps-23* mutants were obtained using alkaline hypochlorite treatment of gravid adult hermaphrodites that had been kept in optimal temperature and feeding conditions for at least 3 generations. The eggs were placed onto plates containing either *E. coli* OP50 or *E. coli* BW25113 and maintained at 20°C. Lifespan measurements were initiated by transfer of L4-stage worms (day 0) to plates containing bacteria grown for 48 h. Worms were transferred to fresh plates every day during the reproductive period and thereafter, every other day until day 12. Worms that showed severe vulva protrusion or bagging were censored. Survival was monitored at regular time points and worms scored as dead if they did not show any movement when prodded with a platinum wire. Each experimental bacterial condition and worm genotype was independently replicated 3 times with each trial containing approximately 50 animals.

#### Translation measurements in worms using surface sensing of translation (SUnSET) assay

The following protocol allows the study of translation through puromycin incorporation without having an obvious effect on general translation (no abnormalities observed on polysome profiles; Arnold et al., 2014). Approximately 6000 N2 and RPS23 K60R mutants were grown on NGM OP50 plates until reaching the L4 stage. Worms were aged at 20°C for an additional 6 h to reach the young adult stage. Worms were collected in M9 media and incubated with 10x concentrated OP50 (from an overnight culture in LB) plus puromycin at a final concentration of 0.5 mg/mL. Worms were incubated at 20°C for 4 h with regular shaking of 200 rpm. Worms were collected and washed 3 times with M9 to remove puromycin and *E. coli.* Worms were resuspended in CelLytic Lysis Buffer (Sigma) plus 1x cOmplete Mini protease inhibitor (Sigma). Worms were lysed by three freeze thaw cycles and using a Q700 sonicator waterbath (Qsonica) kept at 4°C with 5x15 s pulses at 100% amplitude. Lysates were centrifuged at maximum speed for 30 min at 4°C to pellet cellular debris and the resulting supernatant was transferred to fresh tubes. The protein concentration of each sample was determined using the Bradford assay. Samples were heated at 95°C for 5 min and were loaded into a 4%–20% Criterion TGX precast gel (Bio-Rad) for SDS-PAGE. Separated proteins were transferred onto a nitrocellulose membrane. The membrane was probed with a purified anti-puromycin clone 12D10 primary antibody (Millipore) and an HRP conjugated goat anti-mouse IgG secondary antibody (Sigma-Aldrich) at a 1:1000 and 1:5000 dilution, respectively. The membrane was exposed on film using Clarity Western ECL Substrate (Bio-Rad). The membrane was stripped by immersing in PLUS Western Blot Stripping Buffer (Thermo Fisher Scientific) for 15 min and was reprobed with an anti-tubulin T6074 (Sigma) antibody at a 1:5000 dilution to provide a loading control. Probing with secondary antibody and exposure of the membrane was carried out as before. Membranes were scanned and densitometry was performed using FUJI software (NIH). Bands were detected manually and the background was subtracted from each peak generated. 4 independent biological replicates were performed per condition.

#### CRISPR/Cas9 in *Drosophila*

The gRNA with the best performance in the T7 assay, performed in S2R+ cells, was cloned into the pCFD3U6 vector for fly embryo injections. The gRNA oligos were designed to have 5′ GTCG-N19/20 in the sense oligo and 5′AAAC-N19/20 reverse complement in the antisense oligo, to allow BbsI enzyme cutting. The oligos were annealed and phosphorylated (RPS23 oligos GTCGCTACCGTCACGGGGCACGA and AAACTCGTGCCCCGTGACGGTAG; PNK ligation buffer (NEB; M0201S) and T4 PNK enzyme (NEB; M0201S)). The oligos/inserts were ligated (T4 DNA ligase; NEB) to the pCFD3U6 vector, which was digested by the BbsI enzyme (NEB; R0539). DH5-α bacteria competent cells were used for transformation. Positive colonies were verified by PCR using an insert binding primer GTCGCTACCGTCACGGGGCACGA and a pCDF3U6 binding primer GTTCGCTTAATGCGTATGCA.

The single-strand oligodeoxynucleotide (ssODN) was designed to have around 80 nucleotides surrounding the mutation of interest and have a mutation disrupting the protospacer adjacent motif (PAM) sequence. Mutation in PAM stops the Cas9 from cutting the DNA once the sequence is recombined in the genome. The ssODN also introduced a silent mutation for a novel restriction site (PvuII) allowing easy PCR screening. The silent PvuII mutation was designed by the Watcut website. The ssODN sequence used to introduce the K60R mutation to the RPS23 gene was the following (mutated bases are underlined): ATATGATATCAATTATATTAATCTCTTAGTGGTATATCAAAACTAATCGGTTTCCTCTACTCCACAGCGGCGTCGAGGCCCGCCAGCCCAACTCAGCCATCCGCAAGTGCGTGAGGGTGCAGCTGATCAAGAACGGCAAGAAGATCACCGCCTTCGTGCCCCGTGACGGTAGCTTGAACTACAT.

Both the ssODN and pCFD3U6 vector containing the cloned RPS23 gRNA were injected into the Cas9 overexpressing embryos (BDSC #54591 flies express the Cas9 protein under the nanos GAL4). Injections were performed by the BestGene company.

To screen for positive CRISPR mutants, we took advantage of the PvuII restriction site that was introduced by the ssODN and hence present only in PCR products from positive candidates that also had the K60R mutation. Primer sequences used were CGACAAGGACTACAAGAAGG and TGCTTGTCTGGAAAAAGATT and Taq DNA polymerase (NEB; M0273) was used for PCR. PvuII restriction (NEB; R0151) was performed after PCR, directly in a Taq PCR mixture, without prior DNA purification. The PCR product size was 646 bp, and upon enzyme restriction the PCR product size was 400 bp and 246 bp for positive RPS23 K60R candidates. In addition, for screening, we also used primers that discriminate between wild-type RPS23 and RPS23 K60R, and primer sequences were GTCCGAAAATCGCACAAAATCCAG and GGCTGAGTTGGGCTGGCG; for PCR condition details please see below. Finally, positive candidates were confirmed by sequencing.

#### Backcrossing of the *Drosophila* RPS23 K60R mutant

CRISPR/Cas9 generated RPS23 K60R mutants were backcrossed before experimental assays. In the first cross, *w*^*Dah*^ virgin females were mated with RPS23 K60R mutant males, to ensure that mitochondria in the crossed strain is passed on from the *w*^*Dah*^ background, enabling the adequate comparison with the control flies for lifespan and other experimental assays. Next, approximately 60 to 80 single fly crosses between a RPS23 K60R mutant female and a *w*^*Dah*^ male were performed in each round of backcrossing, and females were then sacrificed and verified by PCR for RPS23 K60R mutant status. For the PCR, genomic DNA was extracted by adding 50 μl of Squishing Buffer (10 mM Tris HCl pH8 (Affymetrix; 22638)); 1 mM EDTA (Sigma; EDS-100G); 25 mM NaCl (Sigma; S3014)) with 0.2 mg/mL proteinase K (Biotechnology; E195). The individual flies were mashed and incubated for one h at 37°C and then the proteinase K was inactivated at 95°C for 15 min. PCR primers were designed to amplify only RPS23 mutant sequence using Taq polymerase under the following conditions: 95°C 3 min denaturation, then 33 cycles of 95°C 30 s, 60°C 15 s, 68°C for one minute, and a final extension of 68°C for 5 min. Primer sequences used were GTCCGAAAATCGCACAAAATCCAG and GGCTGAGTTGGGCTGGCG, resulting in a PCR product 692bp long if flies contained the RPS23 K60R mutation and no amplification for the wild-type flies. Virgin fly progeny was collected for the next round of backcrossing only from vials in which parental females were positive for RPS23 K60R mutation and then used to set up the next round of single crosses with *w*^*Dah*^ males. Seven rounds of backcrosses were performed prior to using RPS23 K60R mutant flies in experimental assays.

#### *Drosophila* stocks

RPS23 K60R was generated in this study, as described above, by CRISPR/Cas9. Dual luciferase reporters were also generated in this study as described below. Stop codon readthrough and misincorporation reporter, were inserted under ubiquitin promoter to a modified pUASTattB vector and integrated into fly genome using Phi31integrase-mediated site specific trangenesis to attP154 site (*Drosophila* embryo injection performed by BestGene). Other strains were obtained from Bloomington *Drosophila* Stock Center (BDSC) or are described in Flybase: FRT42D (BDSC, 1802 and 5626), *UAS-FLP* (BDSC, 4540), hh-GAL4 (FlyBase, FBti0017278), hs-FLP (Flybase, FBtp0000267). In Figures 1I and S3F genotype was: hs-FLP/+; FRT42D RPS23^K60R^ /FRT42D ubi-GFP. In Figure S3G genotype was: FRT42D RPS23^K60R^/FRT42D ubi-GFP; hh-GAL4, UAS-FLP/+.

#### *Drosophila* longevity assays

RPS23 K60R heterozygous flies for longevity assays were obtained by backcrossing to standard *w*^*Dah*^ background and confirmed by PCR. For all experiments, flies were reared at standard larval density by transferring 18 μl of egg suspension into SYA bottles. Eclosing adults were collected over a 12 h period and allowed to mate for 48 h before sorting into single sexes and placed in vials containing either control or experimental drug food. For lifespan assays, flies were reared at standard density and maintained at 15 flies per vial. Flies were transferred to fresh food vials every 2-3 days and scored for deaths. At least 150 flies were used for each lifespan experiment.

#### Western blot measurements in *Drosophila*

Flies were homogenized in 2x Laemmli loading sample buffer (100 mM Tris pH 6.8, 20% glycerol, 4% SDS; Bio-Rad; 1610747) containing 50 mM DTT, protease inhibitor (cOmplete Mini EDTA-free; Roche) and phosphatase inhibitor (PhosSTOP EASYpack; Roche) cocktails. Extracts were cleared by centrifugation and approximately 20 μg of protein extract was loaded per lane on a polyacrylamide gel. Proteins were separated and transferred to nitrocellulose membrane using wet transfer. The following antibodies were used at the indicated dilutions: H3 (Cell Signaling Technology; 4499S; 1:2000), pS6K (Cell Signaling Technology; 9206S; 1:1000), total S6K (kind gift from Linda Partridge laboratory; 1:1000), p4EBP (Cell Signaling Technology, 2855S; 1:500), non-phospho4E-BP (Cell Signaling Technology; 4923S; 1:500), peIF2α (Cell Signaling Technoology; 3398S; 1:1000), eIF2S1(abcam; ab26197; 1:1000); GAPDH (GeneTex; #GTX100118; 1:2000); anti-puromycin [3RH11] (Kerafast; Equation 0001; 1:2000). Blots were developed using the ECL detection system (Clarity Western ECL Substrate; Bio-Rad; 1705060), imaged using CCD camera system of ImageQuant LAS 4000 (GE Healthcare), and analyzed using FIJI software (US National Institutes of Health). We used precasted Any kD TGX stain-free gels (Bio-Rad; 567-8123 or 567-8124) according to the manufacturer’s instructions. Total proteins were imaged using Stain-Free Imaging Bio-Rad technology upon one minute UV activation step using Bio-Rad ChemiDoc system, and by Ponceau S staining of the membrane.

#### Development time in *Drosophila*

Eggs were collected from flies in cages onto grape juice agar plates over a defined time window (< 4 h). After ∼24 h, the resulting L1 larvae were picked onto SYA food at a density of 50 per vial (n > 150 total per genotype), and the time to pupariation and adult eclosion was monitored.

#### Negative geotaxis or climbing assay in *Drosophila*

For this assay, which was performed once per week, 15 adult flies were placed in a vertical column consisting of two vials separated by the Drosoflipper device (http://www.drosoflipper.com/). Flies were tapped to fall on the bottom of the vials and climbing was monitored for 45 s. Flies reaching the top of the column or remaining at the bottom after a 45 s period were counted. Each cohort was evaluated three times and 10 groups of 15 flies were used per genotype. The mean number of flies at the top (*n*top), the mean number of flies at the bottom (*n*bottom) and the total number of flies assessed (*n*tot) were recorded. Performance index was calculated as 1/2(*n*tot + *n*top −*n*bottom)/*n*tot, as described in Rogers et al. (2012).

#### Heat shock stress assays in *Drosophila*

Flies in batches of 15 were placed in empty vials (n = 3 to 6 batches per genotype). These vials were placed in a water bath at 39°C for 30 min period during which all flies were knocked down, which was scored. Flies were then transferred to fresh vials and recovered flies were scored the next day and the percentage of survival calculated.

#### Fecundity assays in *Drosophila*

Flies were let to lay eggs in vials beetween 8 and 48 h, depending on the age of the flies and egglaying output. Eggs were counted once per week from 10 vials per genotype, each vial containing 15 flies. The total number of eggs laid per fly per day was calculated.

#### RNA extraction, cDNA, RT-qPCR in *Drosophila*

For RNA extraction we used dissected heads and thoraxes from ten 10-day-old flies. Total RNA was isolated using TRIZOL (ThermoFisher Scientific; 15596026). Samples were homogenized in a Ribolyser (FastPrep Classic; MP Biomedicals). RNA pellets obtained were resuspended in 20 μL of nuclease-free water (ThermoFisher Scientific; AM9937). Concentration and purity were determined using a NanoDrop spectrophotometer (ThermoFisher Scientific). The RNA samples were stored at −80°C. DNA was degraded using the Ambion DNase I kit (ThermoFisher Scientific; AM2222). RNA was converted to cDNA using random hexamers and ProtoScript II Enzyme mix (New England Biolabs; E6560S). Quantitative PCR was performed using Power SYBR Green PCR Master Mix (ThermoFisher Scientific; 4367659), using the relative standard curve method on an Eppendorf Realplex Mastercycler. Primer sequences used are: for actin5C GAGCGCGGTTACTCTTTCAC and GCCATCTCCTGCTCAAAGTC; for rps23 CGCTTCAAGGTTGTCAAGGT and AGATCTTGGGCGTTCCTTCT.

#### Relative translation rates in adult *Drosophila*

Translation was measured with the SUnSET assay (Deliu et al., 2017; Filer et al., 2017; Schmidt et al., 2009) that is based on the incorporation of puromycin, which is a tRNA analog, into newly-synthesized peptides, allowing their detection by immunoblotting using anti-puromycin antibody [3RH11] (Kerafast; 1:2000; Equation 0001). Three intestines were dissected from 10-day-old flies in Schneider’s medium and transferred simultaneously to Schneider’s medium containing puromycin at 10 μM (Sigma; P8833) for a 30 min incubation in a ThermoMixer at 25°C with gentle shaking. The reaction was stopped by snap-freezing in dry ice and stored at −80°C. Subsequently, samples for western blot analysis were prepared using our standard method described above.

#### Generation of clones by FLP-FRT-mediated mitotic recombination in *Drosophila* larvae

Mitotic recombination clones were generated by the FLP-FRT technique (Germani et al., 2018). We introduced an FRT site proximal to RPS23 K60R mutation. In the presence of an FRT site in *trans* at the same location in the homologous chromosome, FLP recombinase induced mitotic recombination. This led to generation of clones, which were either homozygous for RPS23 K60R or wild-type. We used heat shock inducible flipase (hs-flp), in which case cells surrounding mitotic clones were heterozygous for RPS23 K60R. We also used hedgehog GAL4, which induced expression of flipase only in the posterior part of the wing imaginal disc (hh-GAL4 > UAS-flp), in which case the entire anterior part of the wing imaginal disc remained heterozygous for RPS23 K60R, while the posterior part was formed of both wild-type and RPS23 K60R homozygous clones. For FLP-FRT experiments, flies layed eggs for 4 h. If hs-flp was used, then larvae at the L2 and L3 stage were heat shocked for 1 h at 37°C using a water bath. 24 h after the second heat-shock larvae were dissected and immunostained. In case of hh-GAL4 > UAS-flp, pre-wandering L3 larvae were dissected and immunostained.

#### Translation measurements by puromycin incorporation assay in *Drosophila* larval wing imaginal discs

To measure translation in larvae we used an alkyne analog of puromycin, O-propargyl-puromycin (OPP) (Liu et al., 2012), which incorporates into the C terminus of translating polypeptide chains thereby stopping translation. This leads to C-terminal alkyne labeled truncated proteins, which can be detected via Cu(I)-catalyzed click chemistry. We used Azides of fluorescent dyes to visualize proteins. For this click chemistry labeling, larvae were inverted in prewarmed Schneider’s *Drosophila* Medium at 25°C (GIBCO, Thermo Fisher Scientific 21720024) and transfered to 1.5 mL Eppendorf tube containing Schneider’s *Drosophila* Medium with 10 μM OPP (Jena Bioscience; NU-931-05, 20mM stock in DMSO). Incubation was at 25°C for 20 min with gentle rocking. Upon washing in PBS, samples were fixed in 4% methanol-free formaldehyde for 20 min and then washed/permeabilized in PBS with 0.2% Triton X-100 for 15 min, both at room temperature with gentle rocking. Incubation with the Click reaction mixture (PBS with 2.5 μM picolyl Azide Alexa Fluor 594, 0.1 mM THPTA, 2mM fresh Na Ascorbate and 1mM CuSO4) was performed for 30 min in the dark at room temperature. Upon washing in PBS with 0.2% Triton X-100, wing discs were dissected out and mounted in Vectashield mounting media with DAPI (Vector Laboratories; H-1200). Wing discs were imaged by confocal miscroscope Zeiss LMS880.

#### Translation fidelity dual luciferase assays for *in vivo* in *Drosophila*

For *in vivo* translation fidelity measurements in flies, we adapted a protocol using dual luciferase yeast constructs that were previously published (Kramer et al., 2010; Salas-Marco and Bedwell, 2005). These translation fidelity reporters were cloned in the modified pUAST-attB vector, where the UAS sequence was replaced by either hsp70 or ubiquitin promoters, and were injected in fly embryos by the BestGene company. The presence of the correct construct was verified by PCR using primers that gave a 1048 bp product (GGAAGATCTATGACTTCGAAAGTTTATGATCCAG and GCCTTATGCAGTTGCTCTCC). These reporters are based on a *Renilla* luciferase followed by a firefly luciferase that are separated by an in-frame linker sequence that results in expression of both luciferases (Kramer et al., 2010; Salas-Marco and Bedwell, 2005). The linker sequence codes for a sense codon in the control construct, and a stop codon followed by a C (UGA*C*) in the stop codon readthrough construct. For misincorporation measurements we use a reporter with a mutation in the active site of firefly H245K (CAC245CGC). In all cases, *Renilla* luciferase was used for normalization of the level of mRNA abundance and translational efficiency (Kramer et al., 2010; Salas-Marco and Bedwell, 2005). The percentage stop codon readthrough was calculated by dividing firefly/*Renilla* ratio of the stop codon readthrough or misincorporation reporter by the average firefly/*Renilla* of the control reporter, as in previously published literature (Kramer et al., 2010; Salas-Marco and Bedwell, 2005).

For luciferase assays, we used the Dual Luciferase Assay Reporter Assay System (Promega; E1910). Four flies per sample were mashed in 35 μL of 1x passive lysis buffer (PLB) (Promega; E1910) and left shaking for 4 h at room temperature. 30 μL of each sample was transferred to a 96 well white microplate (Greiner Bio-one; 655074) leaving an empty well between samples to avoid signal cross-talk contamination and measured using a Varioskan LUX microplate reader (ThermoFisher Scientific; VL0L0TD0).

#### Translation fidelity dual luciferase assays for *in vitro Drosophila* S2R+ cells

The translation fidelity reporters for *Drosophila* S2R+ cells were adapted from yeast (Salas-Marco and Bedwell, 2005). The dual luciferase reporters, for either stop codon readthrough or misincorporation, were obtained from the Bedwell lab (pDB686, pDB868, pDB690 and pDB691) (Salas-Marco and Bedwell, 2005). For both *in vivo* and *in vitro* measurements the same contructs were used but adapted by cloning into different vectors. For cell culture experiments these constructs were inserted into the pENTR3C vector using pENTR Directional TOPO Cloning kit (Invitrogen; K2400-20) and then transferred to expression vectors containing the copper-inducible metallothionein (pMT) promoter. For a stronger luminescent signal, stable cell lines expressing the translation fidelity reporters were made. Stable cell lines were made following a standard procedure with the pAC5-pCO-Blast plasmid, the Effectene Transfection Reagent kit (QIAGEN; 301425), and Blasticidin selection (ThermoFisher Scientific; A1113903; 30 μg/mL).

S2R+ cell number and viability were measured using the Countess II Automated Cell Counter (ThermoFisher Scientific; AMQAX1000). 100,000 cells in 500 μL Schneider medium with 10% heat-inactivated FBS and penicillin G (ThermoFisher Scientific; BP2955-5) were seeded onto 48 well plates (Corning Costar; 3548). After 2 days, drugs were added at the indicated concentration together with CuSO_4_ (Sigma; I2852; 500 μM) to induce luciferase expression. Drugs added to the cells include paromomycin sulfate salt (Sigma; P5057; 50 mM stock in H_2_O); rapamycin (LC Laboratories; 4 mM stock in ethanol), trametinib (LC Laboratories; 5 mM stock in DMSO), Torin 1 (Tocris; 1 mM stock in DMSO) and incubated for 16 h at 25°C. To prepare the samples for the dual luciferase assay, the plates were centrifuged and pellets washed with PBS, followed by pellet freezing at −80°C for 30 min to enhance lysis. Upon addition of 20 μL of 1x PLB from the Dual-Luciferase Reporter Assay System kit (Promega; E1910), plates were incubated at room temperature for 15 min. Once lysed, 10 μL of sample was transferred to a white 96 well plate and read using the Varioskan LUX Microplate Reader and the Dual-Luciferase Reporter Assay System kit reagents LARII and Stop&Glo.

### QUANTIFICATION AND STATISTICAL ANALYSIS

Statistical analysis was performed using JMP (version 14.0.5; SAS Institute) and Prism 8 software. Log-rank tests were performed on lifespan curves. Data were expressed as means ± SEMs in figures and text. Paired or unpaired two-tailed t tests were performed as appropriate. One-way or two-way ANOVA were used to make comparisons across more than two groups, with either Tukey’s or Sidak’s multiple comparison test was used. The statistical parameters for each experiment can be found in the figures and figure legends. In figures, asterisks denote statistical significance as (^∗^p < 0.05, ^∗∗^p < 0.01, ^∗∗∗^p < 0.001, ^∗∗∗∗^p < 0.0001) as compared to appropriate controls.

## Data Availability

All data reported in this paper will be shared by the lead contact upon reasonable request. Computer codes used in this study are available from GitHub: https://github.com/Cabreiro-Lab/cell-metab.phylo and https://github.com/saulmoore1/PhD_Project.git. Any additional information required to reanalyze the data reported in this paper is available from the lead contact upon request.

## Supplemental information

Document S1. Figures S1–S6

Table S1. Environmental data of archaea presented in the phylogenetic tree of the archaea kingdom and used for the MSA and HCPC analyses, related to Figures 1, 2, and S2

Table S2. Summary table of 2,090 worm behavioral features and 177 significant features for RPS23 K60R mutant compared to wild-type N2, related to Figures 2 and S4

Document S2. Article plus supplemental information
