# Supplementary material for: Increased fidelity of protein synthesis extends lifespan
Source: Cell Metab. 2021 Nov 2;33(11):2288–2300.e12. doi: 10.1016/j.cmet.2021.08.017 (PMC8570412; doi:10.1016/j.cmet.2021.08.017)
Supplement: Document S1. Figures S1–S6 [file mmc1.pdf]

Cell Metabolism, Volume *33*

Supplemental information

Increased fidelity of protein synthesis extends lifespan

Victoria Eugenia Martinez-Miguel, Celia Lujan, Tristan Espie–Caullet, Daniel Martinez-Martinez, Saul Moore, Cassandra Backes, Suam Gonzalez, Evgeniy R. Galimov, André E.X. Brown, Mario Halic, Kazunori Tomita, Charalampos Rallis, Tobias von der Haar, Filipe Cabreiro, and Ivana Bjedov

Increased fidelity of protein synthesis extends lifespan

Victoria Eugenia Martinez-Miguel, Celia Lujan, Tristan Espie--Caullet, Daniel Martinez-Martinez, Saul Moore, Cassandra Backes, Suam Gonzalez, Evgeniy Galimov, André E. X. Brown, Mario Halic, Kazunori Tomita, Charalampos Rallis, Tobias von der Haar, Filipe Cabreiro*, Ivana Bjedov*

*Correspondence to: i.bjedov@ucl.ac.uk, f.cabreiro@imperial.ac.uk

This PDF file includes:
Supplementary Figures S1 to S6

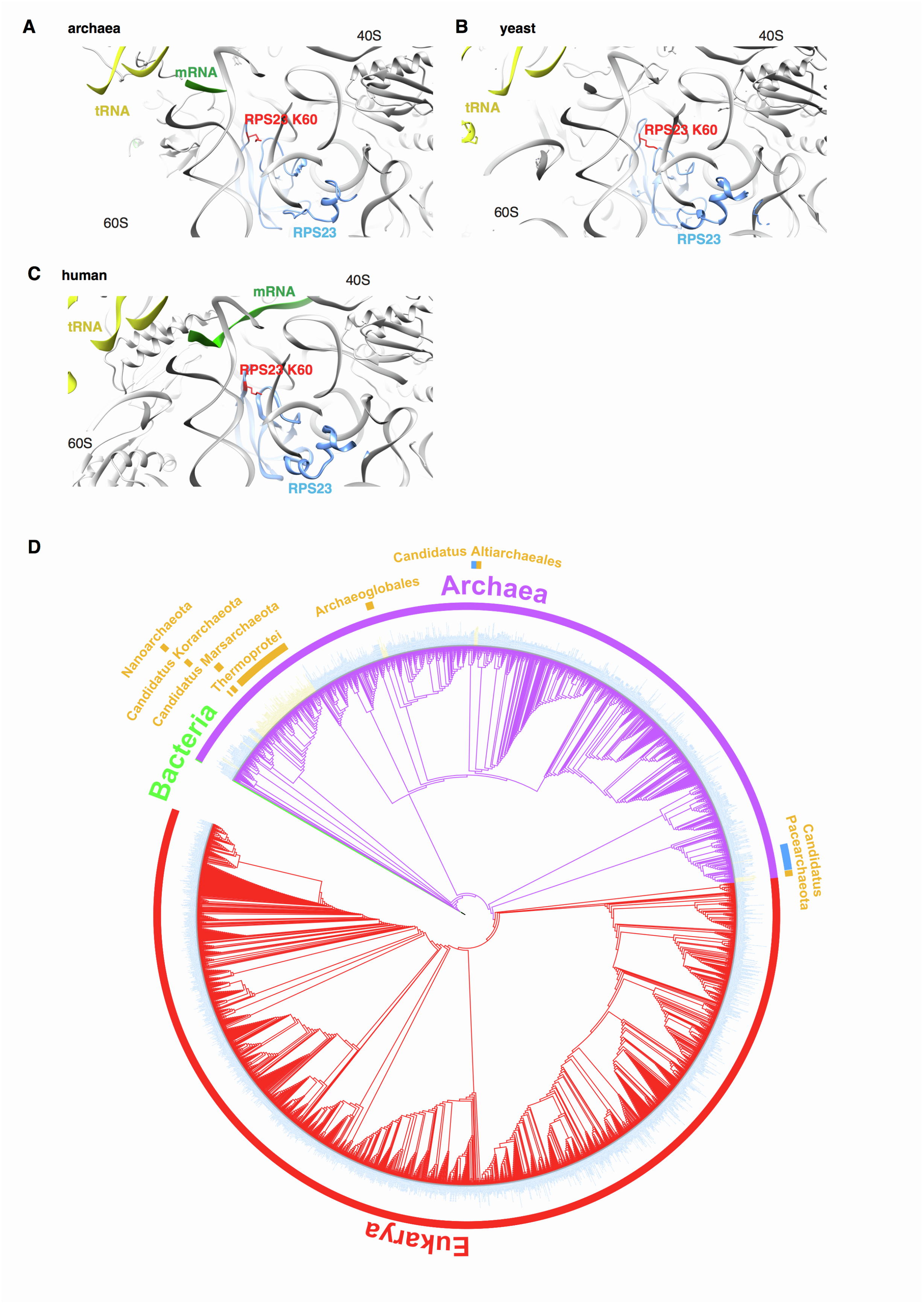

A
archaea
40S
mRNA
tRNA
RPS23 K60
60S
RPS23
B
yeast
40S
tRNA
RPS23 K60
60S
RPS23
C
human
40S
mRNA
tRNA
RPS23 K60
60S
RPS23
D
Candidatus Altiarchaeales
Archaea
Archaeoglobales
Nanoarchaeota
Candidatus Korarchaeota
Candidatus Marsarchaeota
Thermoprotei
Bacteria
Candidatus Pacearchaeota
Eukarya

Figure S1. Phylogenetic tree of the ribosomal protein S12/S23 protein sequences from archaea and eukarya domains, without branch lengths. Related to Figure 1. S12 is the ribosomal protein from the small ribosomal subunit and is known as S12 in bacteria, S23 in eukaryotes, and either as S12 or S23 in archaea. **(A-C)** A close-up view of the ribosome decoding center from archaea (*Pyrococcus abyssi*) **(A),** yeast (*Saccharomyces cerevisiae*) **(B)**, and human (*Homo sapiens*) **(C)**, showing RPS23, lysine residue RPS23 K60, tRNA and mRNA. **(D)** *Escherichia coli* is used as the outgroup of the tree. The different colours in the outside ring represent the three life domains. The organisms with names in blue have lysine in the conserved KQPNSA region of the RPS23, while organism with names in orange have arginine instead. The phyla of the organisms with the R variation are represented in yellow in the outer ring and only found in certain Archaea. The archaeal RPS12 is the homologue of RPS23 in eukaryotes. Sequences downloaded from Interpro.

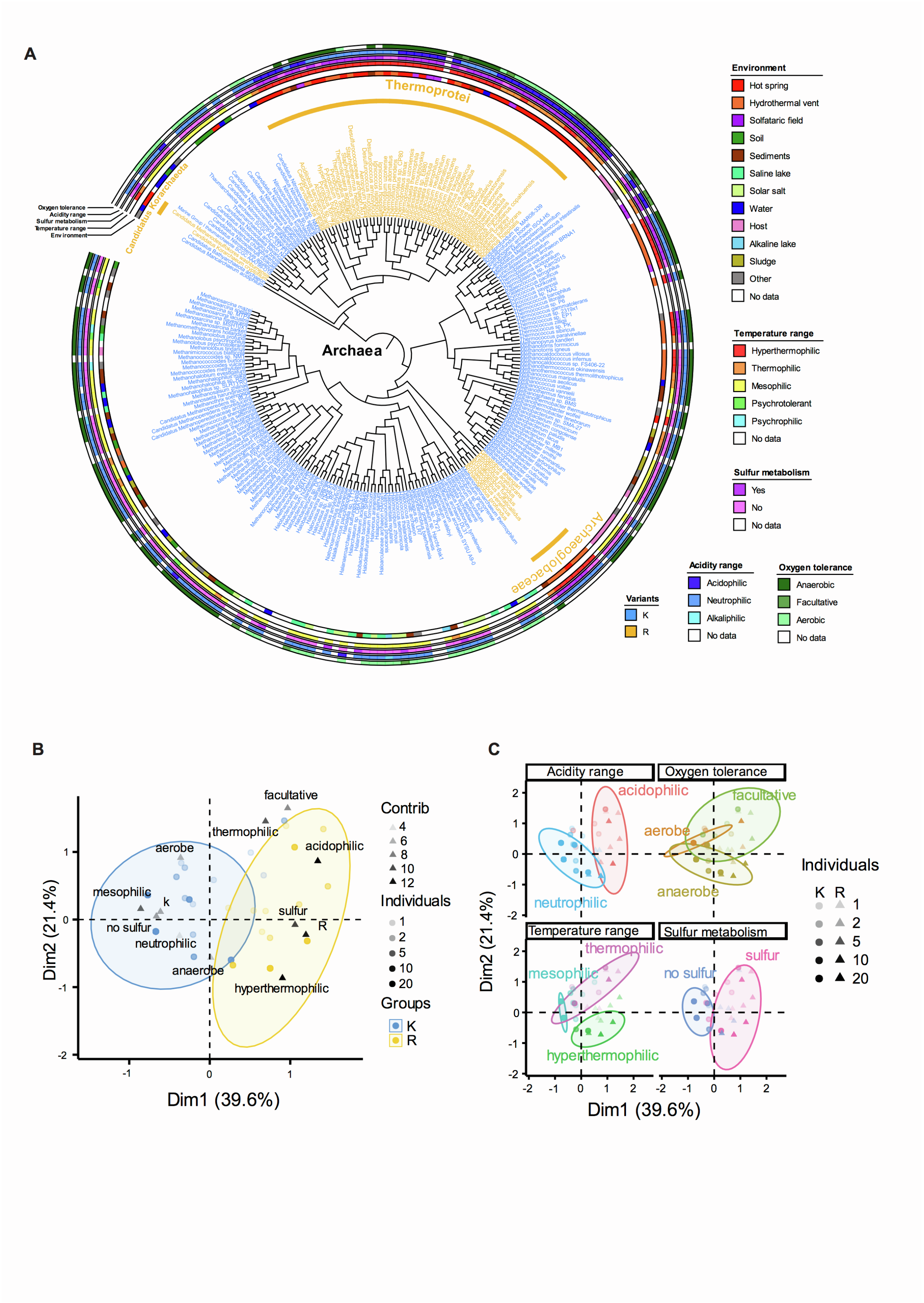
A
Thermoprotei
Candidatus Korarchaeota
Archaea
Archaeoglobaceae
Oxygen tolerance
Acidity range
Sulfur metabolism
Temperature range
Environment
Environment
Hot spring
Hydrothermal vent
Solfataric field
Soil
Sediments
Saline lake
Solar salt
Water
Host
Alkaline lake
Sludge
Other
No data
Temperature range
Hyperthermophilic
Thermophilic
Mesophilic
Psychrotolerant
Psychrophilic
No data
Sulfur metabolism
Yes
No
No data
Variants
K
R
Acidity range
Acidophilic
Neutrophilic
Alkaliphilic
No data
Oxygen tolerance
Anaerobic
Facultative
Aerobic
No data
B
facultative
thermophilic
acidophilic
aerobe
mesophilic
k
sulfur
no sulfur
R
neutrophilic
anaerobe
hyperthermophilic
Dim1 (39.6%)
Dim2 (21.4%)
Contrib
4
6
8
10
12
Individuals
1
2
5
10
20
Groups
K
R
C
Acidity range
Oxygen tolerance
acidophilic
facultative
aerobe
neutrophilic
anaerobe
Temperature range
Sulfur metabolism
thermophilic
sulfur
mesophilic
no sulfur
hyperthermophilic
Dim1 (39.6%)
Dim2 (21.4%)
Individuals
K R
1
2
5
10
20

Figure S2. K and R variants of the ribosomal protein S12/S23 through phylogenetic trees of the archaea kingdom. Related to Figure 1. (A) NCBI taxonomy based phylogenetic tree using the PhyloT web tool. 3 clades containing R variants within the archaea kingdom were identified, the *Thermoprotei* class within the *Crenarchaeota* phylum, the *Candidatus Korarchaeota* phylum and the *Archaeoglobaceae* family within the *Euryarchaeota* phylum. Sequences were from the NCBI Reference Sequence protein database. K and R archaea species are coloured in blue and yellow, respectively. Additional environmental data are shown, from the inside to the outside, the rings show the environment, the temperature range, the dissimilatory sulfur metabolism, the acidity range and the oxygen tolerance for each species. Categories are presented in the legend. Visualization and annotation of the trees performed using iTOL web tool (*2*). **(B)** Maps resulting from the multiple correspondence analysis (MCA) of the archaea and environmental data using the MCA function of the FactoMineR RStudio package. Variables used in this analysis: variant: K, R; temperature range: hyperthermophilic, thermophilic, mesophilic; oxygen tolerance: aerobe, anaerobe, facultative; acidity range: acidophilic, neutrophilic; sulfur metabolism: no sulfur, sulfur. Categorical variables are represented by triangles, transparency represents the contribution to the construction of the plan. Dots represent individuals, transparency represents the number of individuals. K and R species are coloured in blue and yellow respectively. **(C)** MCA maps obtained using same methods as above. K and R species are represented by dots and triangles respectively, transparency represents the number of individuals. Ellipses represent the shape of individual groups for each category variable and are drawn according to a multivariate t-distribution.

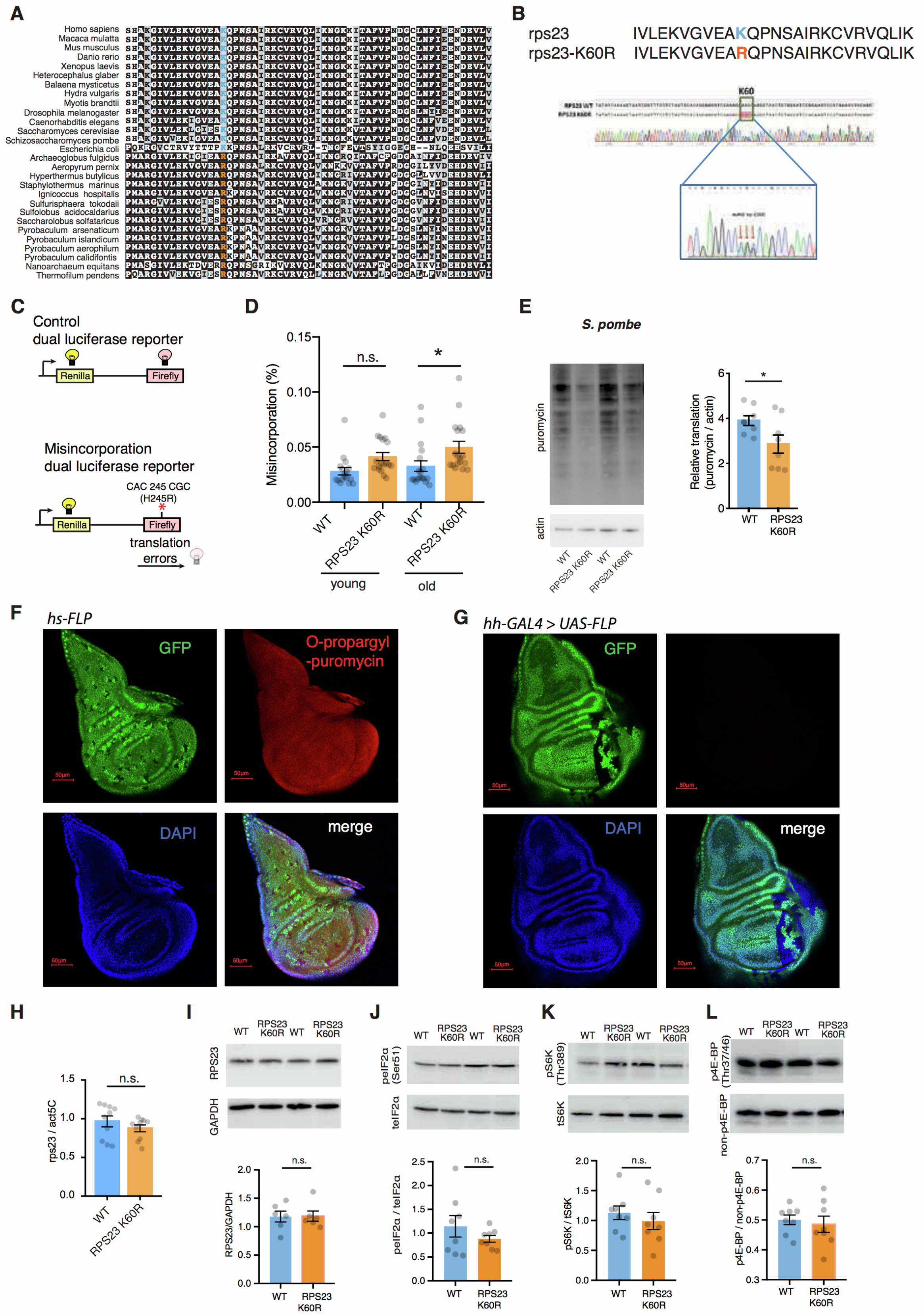
A
B
rps23 IVLEKVGVEAKQPNSAIRKCVRVQLIK
rps23-K60R IVLEKVGVEARQPNSAIRKCVRVQLIK
K60
C
Control
dual luciferase reporter
Renilla
Firefly
Misincorporation
dual luciferase reporter
CAC 245 CGC
(H245R)
translation
errors
D
Misincorporation (%)
n.s.
*
WT
RPS23 K60R
young
old
E
S. pombe
puromycin
actin
Relative translation
(puromycin / actin)
F
hs-FLP
GFP
O-propargyl
-puromycin
DAPI
merge
G
hh-GAL4 > UAS-FLP
GFP
DAPI
merge
H
rps23 / act5C
I
RPS23
GAPDH
RPS23/GAPDH
J
peIF2α
(Ser51)
teIF2α
peIF2α / teIF2α
K
pS6K
(Thr389)
tS6K
pS6K / tS6K
L
p4E-BP
(Thr37/46)
non-p4E-BP
p4E-BP / non-p4E-BP

Figure S3. KQPNSA region of RPS23 is evolutionary conserved and K60R mutation does not impact protein synthesis nor misincorporation errors of RPS23 K60R mutants in *Drosophila*. Related to Figure 1. **(A)** Amino acid sequence alignment of a highly conserved region of the RPS23 orthologues from several model organisms. Identities are marked with black boxes. The QPNSA region is preceded by either a lysine (organisms with K in blue) or arginine (organisms with R in orange). The arginine variant is only found in certain archaea and it is not present in long-lived organisms such as the naked mole rat (*Heterocephalus glaber*), the bowhead whale (*Balaena mysticetus*), the hydra (*Hydra vulgaris*), and the Brandt's bat (*Myotis brandtii*). Sequences taken from NCBI database and from the bowhead whale genome resource. **(B)** DNA sequencing results of the *Drosophila* CRISPR/Cas9 mutants showing K60R mutation. **(C)** Shematic representation of the dual luciferase reporter for measurement of misincorporation errors *in vivo* in flies. **(D)** No significant difference of misincorporation errors in young wild-type flies (n=17) compared to young RPS23 K60R (n=18) (*P*=0.16; one-way ANOVA). Increased levels of misincorporation errors in the RPS23 K60R mutant compared to control in old flies (*P*=0.036; one-way ANOVA; n=18). No increase in misincorporation errors with age for both genotypes (*P*=0.89 for wild-type and P=0.52 for RPS23 K60R young versus old comparison; one-way ANOVA) **(E)** The level of *de novo* protein synthesis, measured by puromycin incorporation and western blotting, showed that in exponentially grown RPS23 K60R mutant *S. pombe* protein synthesis is downregulated compared to control (p=0.039; two-tailed unpaired *t*-tests; n=8). **(F-G)** Wing imaginal disc with most cells heterozygous for the RPS23 K60R mutation (GFP +/-) and with wild-type (GFP +/+) and RPS23 K60R homozygous (GFP -/-) clones that were generated by mitotic recombination using either heat shock inducible flipase (*hh-flp*) (**F**) or hedgehog-GAL4 driven flipase (*hh-GAL4>UAS-flp*) (**G**) confined to the posterior compartment. Puromycilated peptides stained and visualized (red) upon 20 minutes incubation with 10 μM O-propargyl-puromycin **(F)**. **(G)** Negative control without O-propargyl-puromycin incubation. **(H)** K60R mutation does not affect *rps23* gene expression by QPCR. *actin5C* expression was used as a normalizing control (n=10; two-tailed unpaired *t*-test; *P*=0.2973). **(I)** RPS23 K60R mutation does not affect RPS23 protein levels as shown by western blot (n=6; two-tailed unpaired t-test; *P*=0.9511). GAPDH expression was used as a loading control. **(J-L)** RPS23 K60R mutation does not significantly change the phosphorylation of (**J**) eIF2α (Ser51) compared to wild-type control flies (n=8; two-tailed unpaired *t*-test; P=0.2836). **(K)** S6K (Thr398) in RPS23 K60R mutants compared to wild-type control flies (n=8; two-tailed unpaired *t*-test; P=0.426) or **(L)** Phosphorylation of 4E-BP in RPS23 K60R mutants compared to wild-type control flies (n=8; two-tailed unpaired *t*-test; P=0.6467). Data shown as mean ± SEM. In all figures **P*<0.05; ***P*<0.01; ****P*<0.001; *****P*<0.0001; n.s., not significant.

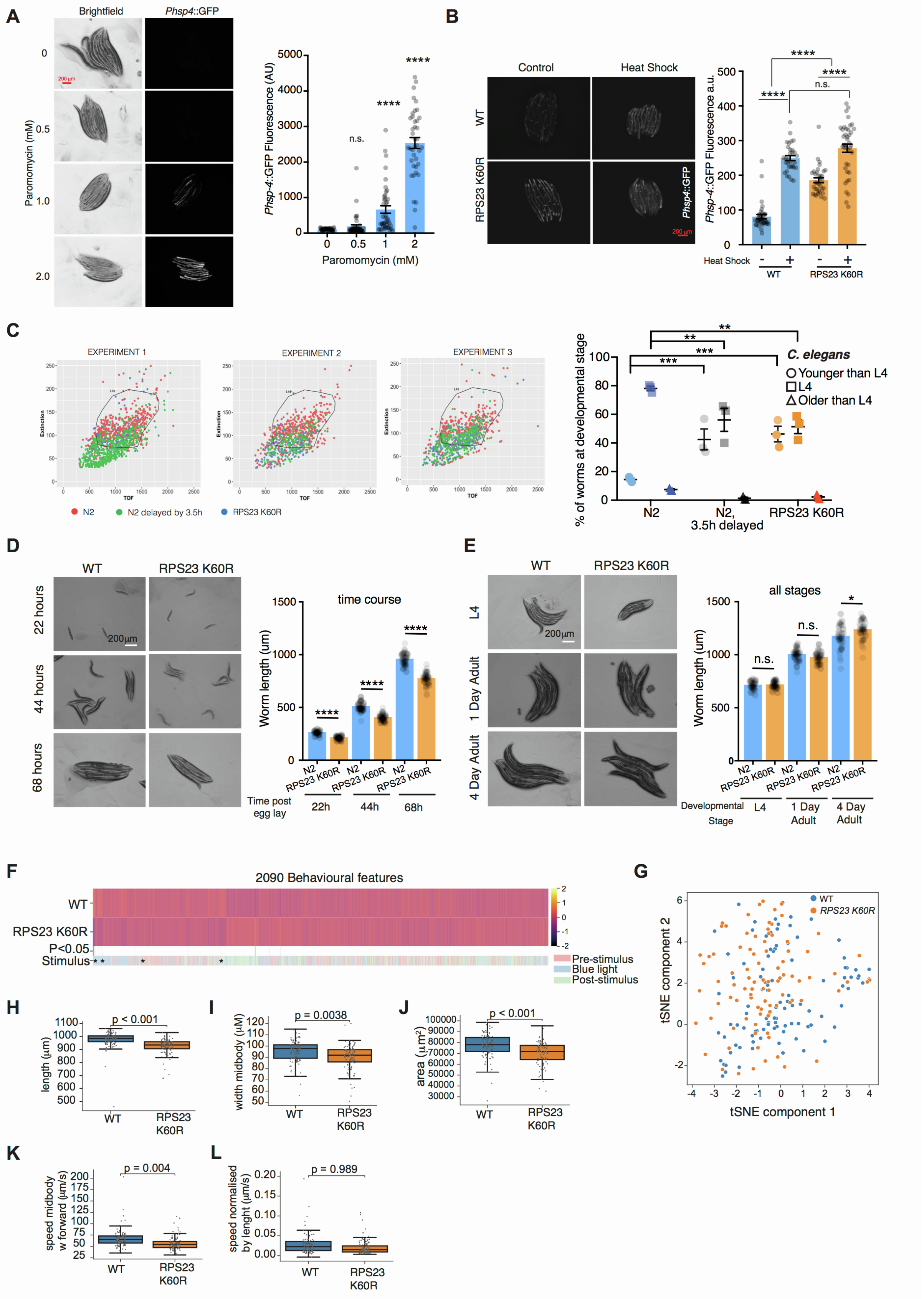
A
Brightfield
Phsp4::GFP
Paromomycin (mM)
0
0.5
1.0
2.0
200 μm
Phsp-4::GFP Fluorescence (AU)
Paromomycin (mM)
n.s.
****
B
Control
Heat Shock
WT
RPS23 K60R
Phsp4::GFP
Phsp-4::GFP Fluorescence a.u.
Heat Shock
WT
RPS23 K60R
n.s.
****
C
EXPERIMENT 1
EXPERIMENT 2
EXPERIMENT 3
Extinction
TOF
N2
N2 delayed by 3.5h
RPS23 K60R
% of worms at developmental stage
C. elegans
Younger than L4
L4
Older than L4
N2
N2, 3.5h delayed
RPS23 K60R
D
WT
RPS23 K60R
22 hours
44 hours
68 hours
200μm
time course
Worm length (um)
Time post egg lay
22h
44h
68h
E
WT
RPS23 K60R
L4
1 Day Adult
4 Day Adult
all stages
Worm length (um)
Developmental Stage
F
2090 Behavioural features
WT
RPS23 K60R
P<0.05
Stimulus
Pre-stimulus
Blue light
Post-stimulus
G
WT
RPS23 K60R
tSNE component 1
tSNE component 2
H
length (μm)
p < 0.001
I
width midbody (μM)
p = 0.0038
J
area (μm²)
p < 0.001
K
speed midbody w forward (μm/s)
p = 0.004
L
speed normalised by lenght (μm/s)
p = 0.989

Figure S4. Developmental delay and morphological phenotypes of RPS23 K60R *C. elegans* mutants. Related to Figure 2. (A) Paromomycin induces a strong up-regulation of endoplasmic reticulum UPR (UPR^{ER}) measured by *Phsp-4*::GFP levels. Each image panel shows 10 individual anaesthetized worms. Each condition on the right represents 4 independent biological replicates with a total of 39-42 worms. Statistical analysis by Brown-Forsythe and Welch ANOVA tests with Dunnett's T3 multiple comparisons tests. **(B)** The RPS23 K60R mutation significantly protects against the effects of heat shock (37°C) on UPR^{ER} activation. Each image panel shows 10 individual anaesthetized worms. Each condition on the right represents 3 independent biological replicates with a total of 31-40 worms. Statistical analysis by 2-way ANOVA with Tukey's comparison test. **(C)** Developmental delay in RPS23 K60R mutants measured by COPAS. The X axis (TOF) is size and Y axis (extinction) which is a measure of the transparency properties of the material. Tested conditions include wild-type N2 controls, wild-type N2 controls delayed by 3.5h egg laying stage synchronisation, and RPS23 K60R mutants. The gating L4 window (black line) was determined by hand-picking worms at the L4 stage. Quantification graph showing the proportion of worms that are in the L4 stage, as well as younger and older worms than L4. Comparison of wild-type with wild-type 3.5h delayed and RPS23 K60R, *P*=0.0008 and *P*=0.0002, respectively. There was a significant decrease in L4 stages for N2 3.5h delayed and RPS23 K60R mutants compared to N2 control worms; *P*=0.0013 and *P*=0.0066, respectively; two-way ANOVA with Tukey's multiple comparison test **(D)** RPS23 K60R mutants are shorter than wild-type worms when compared at defined times post egg-laying. Each image panel on the left shows 4-13 individual anaesthetized worms. Quantification represented on the right. Each condition represents 3 independent biological replicates with a total of 50-58 worms. Statistical analysis by one way ANOVA tests with Sidak correction for multiple comparison **(E)** Anaestesized RPS23 K60R mutants have same length as wild-type worms at the same developmental L4 stage or as 1 day adults, except 4 day adults which are slightly longer than controls. Each image panel on the left shows 4-6 individual anaesthetized worms. Quantification represented on the right. Each condition represents 3 independent biological replicates with a total of 50-54 worms. Statistical analysis by Brown-Forsythe and Welch ANOVA tests with Games-Howell's multiple comparisons tests. Data shown as mean ± SEM. (**F-L**) The RPS23 K60R mutation decreases worm area in young adults but does not alter behavioural features unrelated to size. **F)** Heatmap comparing RPS23 K60R mutant worm behaviour with wild-type N2. Features (columns) are ordered according to hierarchical clustering order for control data with respect to day variation (Z-standardised, metric: 'Euclidean', method: 'complete'). P-values for significant features are depicted with a dark line below the heatmap, along with column colours for the stimulus condition of each feature. Selected features depicted in boxplots are highlighted with an asterisk. **G)** t-distributed stochastic neighbour embedding (tSNE) with 2090 features (perplexity=50). Points are coloured by worm strain. **H)** RPS23 K60R mutant worms are shorter than wild-type. **I)** RPS23 K60R mutant worms are narrower than wild-type. **J)** RPS23 K60R mutant worms are smaller than wild-type. **K)** RPS23 K60R mutant worms are slower than wild-type while moving forwards. **L**) No difference between RPS23 K60R mutant worms and wild-type speed is observed after normalizing by worm length. Measured during blue-light stimulation (**H-J**). (**F-L**) Data shown as mean ± SD. Statistical analysis by linear mixed model and controlling the false discovery rate at 5% with the Benjamini-Yekutieli correction for multiple comparisons.

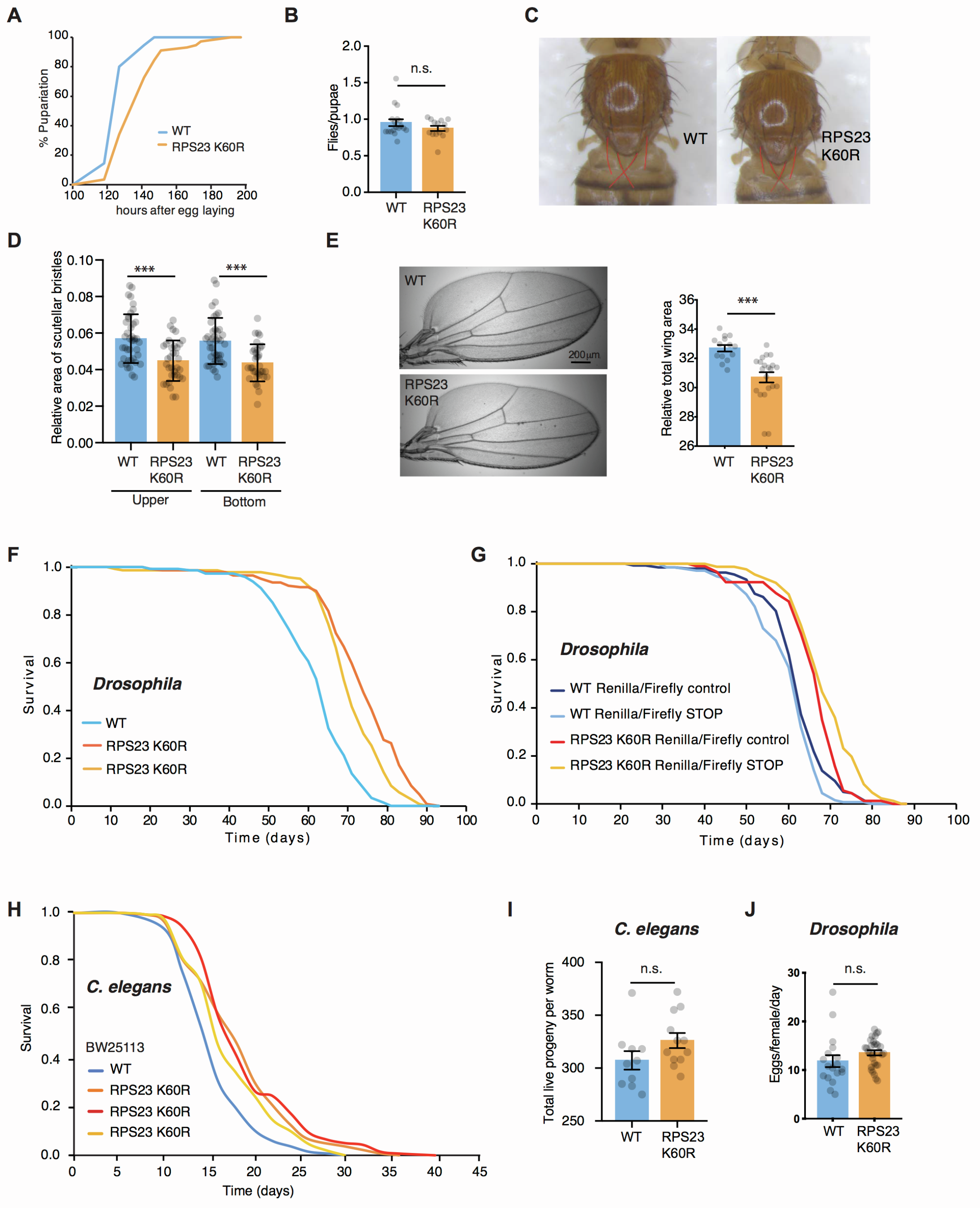

A
% Pupariation
WT
RPS23 K60R
hours after egg laying
B
Flies/pupae
n.s.
WT
RPS23 K60R
C
WT
RPS23 K60R
D
Relative area of scutellar bristles
***
WT
RPS23 K60R
Upper
Bottom
E
WT
RPS23 K60R
200µm
Relative total wing area
***
F
Drosophila
Survival
Time (days)
G
Drosophila
WT Renilla/Firefly control
WT Renilla/Firefly STOP
RPS23 K60R Renilla/Firefly control
RPS23 K60R Renilla/Firefly STOP
H
C. elegans
BW25113
I
C. elegans
Total live progeny per worm
n.s.
J
Drosophila
Eggs/female/day
n.s.

Figure S5. RPS23 K60R mutants are developmentaly delayed and long-lived without impaired fecundity. Related to Figure 2 and Figure 3. (A) Delay in pupariation in RPS23 K60R mutants compared to controls (n~100; log-rank test; p<0.0001). Representative of three independent experiments. **(B)** There was no difference in number of pupae developing into flies between RPS23 K60R and controls (wild-type n=18; RPS23 K60R n=14; two-tailed unpaired *t*-test; *P*=0.211). **(C)** Scutellar bristles of the wild-type flies and the RPS23 K60R at 1 day of age coloured in red using Image J. **(D)** RPS23 K60R mutant has smaller bristles. Comparison between upper and bottom bristles between RPS23 K60R and wild-type flies (wild-type n=40; RPS23 K60R n=34; one-way ANOVA, Tukey's post-hoc test; *P*=0.0002 for both comparisons). **(E)** RPS23 K60R mutant has smaller wings than wild-type flies as measured by Image J. wild-type n=14; RPS23 K60R n=21; two-tailed unpaired *t*-test; *P*=0.0002. **(F)** Independent CRISPR/Cas9 candidates for RPS23 K60R are long-lived compared to WT control. WT compared to RPS23 K60R (orange) (log-rank test; $P=5.08 \times 10^{-24}$) and WT compared to RPS23 K60R (yellow) (log-rank test; $P=2.35 \times 10^{-15}$). **(G)** Longevity analysis of RPS23 K60R mutants and controls bearing dual luciferase stop codon readthrough reporter constructs. RPS23 K60R flies bearing Renilla/firefly control construct were longer-lived than both wild-type flies having Renilla/firefly control construct (n~150; log-rank test; *P*=0.00014) and wild-type flies with Renilla/firefly stop codon readthrough construct (n~150; log rank test; $P=3.3 \times 10^{-9}$). RPS23 K60R flies with Renilla/firefly stop codon readthrough construct were longer-lived than both wild-type flies having Renilla/firefly control construct (n~150; log rank test; $P=2.8 \times 10^{-11}$) and wild-type flies with Renilla/firefly stop construct (n~150; log rank test; $P=8.2 \times 10^{-16}$). **(H)** Longevity of the RPS23 K60R *C. elegans* mutants is independent of the food source. Lifespan analysis of worms grown on BW25113 bacteria as food source is presented. Wild-type N2 controls (dark blue; n=138) are shorter lived compared to either of the RPS23 K60R independent CRISPR/Cas9 lines (orange; n=139; log-rank *P*<0.0001); (red; n=150; log-rank *P*<0.0001); (yellow; n=154; log-rank *P*=0.0005). Representative of three independent trials. **(I)** No signigicant difference observed in total live progeny output between RPS23 K60R mutant and controls (two-tailed unpaired *t*-test; *P*=0.1086). Each genotype represents 3 independent biological replicates with a total of 10-12 worms. **(J)** Cumulative egg laying in *Drosophila* measured over seven-week period shows no difference between RPS23 K60R and control flies (two-tailed unpaired *t*-test; *P*=0.52). Data shown as mean ± SEM; ****P*<0.001; n.s., not significant.

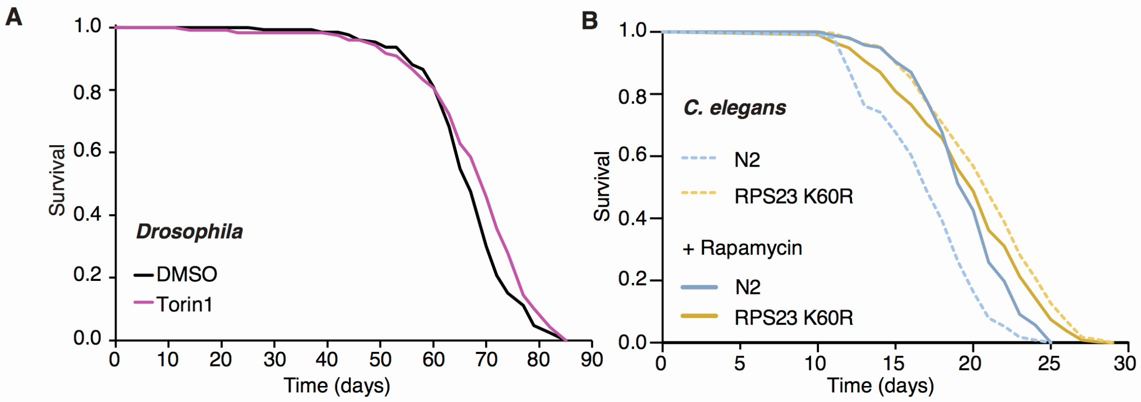

Figure S6. Drug effects on *Drosophila and C. elegans lifespan*. Related to Figure 4. (A) Torin1, a dual catalytic inhibitor of TORC1 and TORC2, extends lifespan in *Drosophila* (n~150; log rank test; *P*=0.037). **(B)** Rapamycin at 100 μM extends lifespan of wild-type *C. elegans* (P<0.001; log-rank test; N2 n=341 and N2 rapamycin n=263). Lifespan of RPS23 K60R was shortened on food supplemented with 100 μM rapamycin. (P=0.0038; log-rank test; RPS23 K60R n=283 and with rapamycin n=334). The survival plot shows the combined survival of 3 independent biological replicates.
